# Enhancer binding kinetics explain transcription factor hub formation

**DOI:** 10.1126/sciadv.aeh1086

**Published:** 2026-10-09

**Authors:** Samantha Fallacaro, Manya Kapoor, Lillian Encarnation, Apratim Mukherjee, Meghan A. Turner, Hernan G. Garcia, Mustafa Mir

**Affiliations:** ^1^ Department of Cell and Developmental Biology, Perelman School of Medicine, University of Pennsylvania, Philadelphia, PA 19104, USA.; ^2^ Developmental, Stem Cell, and Regenerative Biology Graduate Group, Perelman School of Medicine, Philadelphia, PA 19104, USA.; ^3^ Center for Computational and Genomic Medicine, Children’s Hospital of Philadelphia, Philadelphia, PA 19104, USA.; ^4^ Department of Bioengineering, University of Pennsylvania, Philadelphia, PA 19104, USA.; ^5^ Department of Biology, University of Pennsylvania, Philadelphia, PA 19104, USA.; ^6^ Biophysics Graduate Group, University of California at Berkeley, Berkeley, CA 94720, USA.; ^7^ Department of Molecular and Cell Biology, University of California, Berkeley, CA 94720, USA.; ^8^ Department of Physics, University of California, Berkeley, CA 94720, USA.; ^9^ Institute for Quantitative Biosciences-QB3, University of California, Berkeley, CA 94720, USA.; ^10^ Chan Zuckerberg Biohub–San Francisco, San Francisco, CA 94158, USA.; ^11^ Howard Hughes Medical Institute, Children’s Hospital of Philadelphia, Philadelphia, PA 19104, USA.; ^12^ Epigenetics Institute, University of Pennsylvania Perelman School of Medicine, Philadelphia, PA 19104, USA.

## Abstract

Transcription factors (TFs) form dynamic, high-concentration clusters, condensates, or hubs, proposed to increase TF binding frequency at target enhancers. However, how enhancer sequence shapes hub properties remains unclear. We developed a live imaging–based framework to quantify the spatiotemporal relationship between TF hubs and actively transcribed genes in live *Drosophila* embryos. Examining hubs formed by the TF Dorsal across enhancers with defined binding site composition, we find that hub enrichment and persistence scale with the number of Dorsal binding motifs. However, these hub properties do not predict transcriptional bursts for a given enhancer. Combining quantitative imaging with computational modeling, we show that Dorsal hub formation can be explained by TF-DNA binding kinetics alone. These findings support a model in which TF hubs emerge from enhancer-encoded TF-DNA interactions rather than higher-order regulatory assemblies.

## INTRODUCTION

The spatiotemporal regulation of gene expression during embryonic development depends on the ability of sequence-specific transcription factors (TFs) to find and bind their target sites within the crowded nuclear environment. Live imaging and single-molecule tracking (SMT) experiments have shown that TFs bind their genomic targets transiently, with residence times on the order of tens of seconds (*1*, *2*). The local accumulation of TFs around targets, through the formation of hubs or condensates, has been proposed as a mechanism to increase binding frequency and maintain target occupancy despite short residence times (*1*–*7*). These high-local concentration clusters are further thought to catalyze the recruitment of cofactors and transcriptional machinery to amplify gene activation (*8*–*22*). Here, we sought to address the central question of this model: whether hubs drive TF target occupancy by increasing their binding frequency or whether they are an emergent feature of underlying binding kinetics.

Clusters of TFs are referred to as condensates when they exhibit nonstochiometric accumulations and their biophysical properties are consistent with formation via phase separation mechanisms (*23*–*25*). Under the phase separation model, transcriptional condensate formation depends on the nuclear concentration and sequence properties of condensate constituents. Although substantial work has focused on how intrinsic protein features and concentration thresholds govern condensate formation (*7*), far less attention has been directed toward how genomic context may tune hub properties at individual loci in an in vivo context.

Hub localization to genomic sites is dynamically influenced by a combination of targeting through a TF’s protein-protein and protein-DNA interactions (*11*, *16*, *21*, *26*–*30*). A TF’s locus-specific network of interaction partners also plays a role in generating compositionally and functionally distinct hubs (*27*, *30*, *31*). The nuclear concentration of a TF and the number of TF binding sites at a locus have direct impacts on TF occupancy and transcriptional output (*11*, *32*). Recent work has suggested a spatio-temporal relationship where the proximity of a hub to its regulatory elements can increase transcriptional bursting (*16*, *33*–*35*). If hubs differentially influence TF occupancy at genes, their properties should be highly gene dependent, rather than globally dictated by TF identity and nuclear concentration. On the basis of these observations, we hypothesized that the biophysical properties attributed to hubs, such as their size and stability, are not emergent properties of higher-order phenomena such as phase separation. We reasoned that hub properties instead reflect the molecular scale binding and diffusion kinetics of hub constituents in an enhancer-specific manner.

To investigate whether hubs drive or reflect binding kinetics, we leverage the *Drosophila* dorsoventral patterning system. In this system, the TF Dorsal forms a nuclear concentration gradient and activates distinct sets of genes in a spatially dependent manner along the embryo’s dorsoventral axis (*36*, *37*). Because of its morphogenic nature, Dorsal-regulated gene expression provides a natural framework to determine whether hubs function uniformly or differentially at target genes that are expressed at different Dorsal concentration levels. Previous work in the *Drosophila* embryo has shown that TF hubs containing Bicoid, Zelda, or Dorsal are recruited to specific genomic loci even at low concentrations and suggests that morphogen hubs play an active role in fine-tuning transcriptional responses to spatial gradients (*8*, *9*, *21*, *32*, *38*, *39*). Dorsal clusters have recently been proposed to form in a concentration threshold–dependent manner via liquid-liquid phase separation mechanisms (*40*). However, how these clusters associate with target sites and if their properties are tuned in a site-specific manner has not previously been explored.

Here, we use high-resolution lattice light-sheet imaging to analyze how Dorsal hubs form at and interact with transcriptionally active loci. To enable these insights, we developed a live imaging–based framework that quantifies the spatiotemporal relationship between TF hubs and active transcription sites in vivo. Using this approach, we measured Dorsal hub properties including local Dorsal enrichment and the persistence of hubs at enhancers of *short gastrulation* (*sog*) and *snail* (*sna*) and at synthetic enhancers with varying numbers of Dorsal binding sites. We further used our live imaging data, along with SMT experiments, to parameterize a molecular kinetics model that recapitulates Dorsal hub formation at the enhancers we investigated. Together, our results suggest that TF clustering, or hub formation, primarily reflects enhancer-dependent binding kinetics rather than a distinct regulatory layer that increases binding frequency.

## RESULTS

### Dorsal hub persistence and density scale with nuclear concentration

To investigate the biophysical properties of Dorsal hubs, we performed lattice light-sheet imaging on blastoderm stage *Drosophila* embryos expressing endogenously tagged Dorsal-mNeonGreen in the 13th and 14th syncytial nuclear cycles (nc13 and nc14) (*41*). Dorsal forms a nuclear concentration gradient and is found at high concentrations in ventral nuclei, moderate to low concentrations in lateral nuclei, and is largely excluded from dorsal nuclei (Fig. 1A and movie S1). Dorsal’s nuclear concentration gradient is not persistent and is reestablished after each nuclear division. At the onset of mitosis, the cytoplasmic-nuclear concentration ratio flattens, and after nuclear membrane reformation, the gradient is reestablished through a balance of nuclear import and export during interphase (*37*). We observed that Dorsal hubs are visible as soon as Dorsal accumulates in the nucleus after mitosis and remain visible throughout interphase at nuclei in both ventral and lateral regions (Fig. 1, B and C). Further, hubs form even as the nucleoplasmic Dorsal concentration is diminished in the dorsal-lateral region (fig. S1, A and B, and movie S2), which demonstrates that hub formation does not require high nuclear protein levels.

**Fig. 1.**
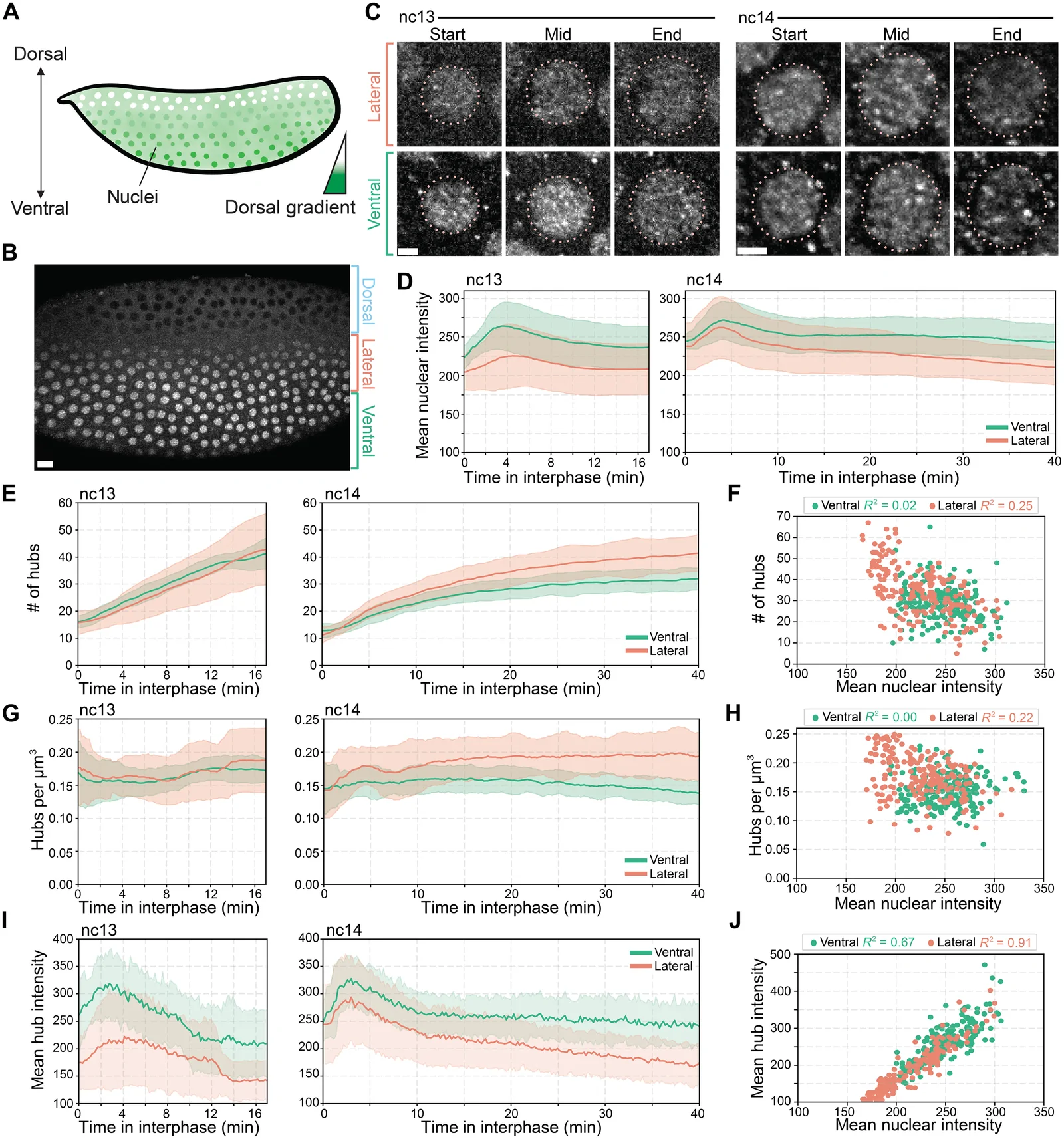
Dorsal hub densities are constant in interphase, but hub enrichment depends on Dorsal nuclear concentration. (**A**) Schematic of the Dorsal gradient in *Drosophila* embryos. Dorsal translocates into ventral surface nuclei at higher rates creating a nuclear concentration gradient along the dorsoventral axis. (**B**) Maximum intensity projection image showing nuclear gradient of Dorsal-mNeonGreen in a *Drosophila* embryo in nc13. Scale bar, is 15 μm. (**C**) Representative single z-slice images of Dorsal-mNeonGreen in nuclei at the lateral (top) and ventral (bottom) embryonic surfaces at the start, middle, and end of nc13 (left) and nc14 (right). Scale bars, 2 μm. (**D**) Average nuclear mean intensity in ventral (green) or lateral (orange) nuclei. Shading shows SD between embryo replicates. *N* = 5 nuclei per embryo, three embryo replicates for each axis position. (**E**) Average number of hubs per nucleus in ventral (green) or lateral (orange) nuclei. Shading shows SD between embryo replicates. (**F**) Scatterplot of number of hubs versus mean nuclear intensities. *R*^2^ value is Pearson’s correlation (*N* = 5814 ventral hubs, 6928 lateral hubs from *N* = 3 embryos per axis position). (**G**) Average hub density (number of hubs per cubic micron) in ventral (green) or lateral (orange) nuclei. Shading shows SD between embryo replicates. (**H**) Scatterplot of hub densities versus mean nuclear intensities. *R*^2^ value is Pearson’s correlation. (**I**) Mean intensities of Dorsal hubs in ventral (green) or lateral (orange) nuclei. Solid line shows average intensity, and shading shows SD between embryo replicates. (**J**) Scatterplot showing hub mean intensity and mean nuclear intensity. *R*^2^ is Pearson’s correlation.

To understand how nuclear concentrations influence hub formation, we compared ventral nuclei along the presumptive ventral furrow with nuclei at the lateral surface (Fig. 1B). Here, we define lateral nuclei as the region of the embryo where Dorsal nuclear concentration diminishes to be equivalent to the cytoplasmic concentration (fig. S1, A and B). Nuclear Dorsal intensity in both ventral and lateral nuclei rises for the first 4.5 min of interphase and then falls for the remainder of interphase (Fig. 1D). To compare Dorsal hubs in these nuclei, we developed a custom analysis pipeline to detect hubs and quantify their intensities, spatial distributions, and persistence (fig. S1C and movie S3). While the total number of detected hubs per nucleus increases during interphase (Fig. 1, E and F), this increase is correlated with the expansion of nuclear volume in this time period (fig. S1, D and E). Thus, despite the dynamic change in nuclear concentration during interphase, the density of Dorsal hubs (number of hubs per unit volume) remains relatively constant during the interphases of nc13 and nc14 (Fig. 1, G and H). We suspect that the apparent increase in the hub number is due to tightly spaced accumulations at binding sites becoming resolvable as the nuclear volume expands rather than de novo formation of additional hubs.

Although the density of hubs is uncoupled with nuclear concentration, the average hub intensity is strongly correlated with nuclear Dorsal concentration (Fig. 1, I and J, and fig. S1H). To further assess hub variability using a segmentation-independent metric, we calculated the coefficient of variation (CV) of nuclear Dorsal intensity (fig. S1, F and G). The CV exhibits an intermediate correlation with nuclear concentration likely since the CV reflects intensity dispersion and not just morphological differences. On the basis of these data, we hypothesized that hubs form rapidly at dorsal target sites at the start of interphase, and then increasing amounts of Dorsal interact with these sites as nuclear concentrations increase.

### Dorsal hubs preferentially enrich and persist at target sites

To determine whether Dorsal hubs preferentially form at transcriptionally active target sites, we performed two-color imaging of Dorsal-mNeonGreen with MCP-mCherry to visualize the site of transcription from MS2 reporters. We characterized hub formation at *sna* and *sog* target loci, as well as *hunchback* (*hb*) as a non-Dorsal target control (Fig. 2, A and B, fig. S2, and movies S4 and S5). Both *sna* and *hb* were analyzed using BAC reporters (*42*) with *sna* expressed in ventral nuclei and *hb* as a non-Dorsal target control in both ventral [*hb(v)*] and lateral [*hb(l)*] nuclei. Conversely, *sog* was driven by a transgenic reporter within its lateral expression domain (*43*). These reporters differ substantially in the number and arrangement of Dorsal binding sites with *sna* containing the highest site density (∼16 sites) compared to *sog* (∼4 sites) and *hb* (no Dorsal binding sites) (Fig. 2A and fig. S2). All constructs were integrated into the same genomic docking site on chromosome III, allowing us to examine enhancer grammar as the primary variable. Visually, we found that Dorsal hubs are most stably associated with *sna* and slightly less so at *sog* compared to nonspecific interactions at *hb(v)* and *hb(l)* (movies S4 and S5).

**Fig. 2.**
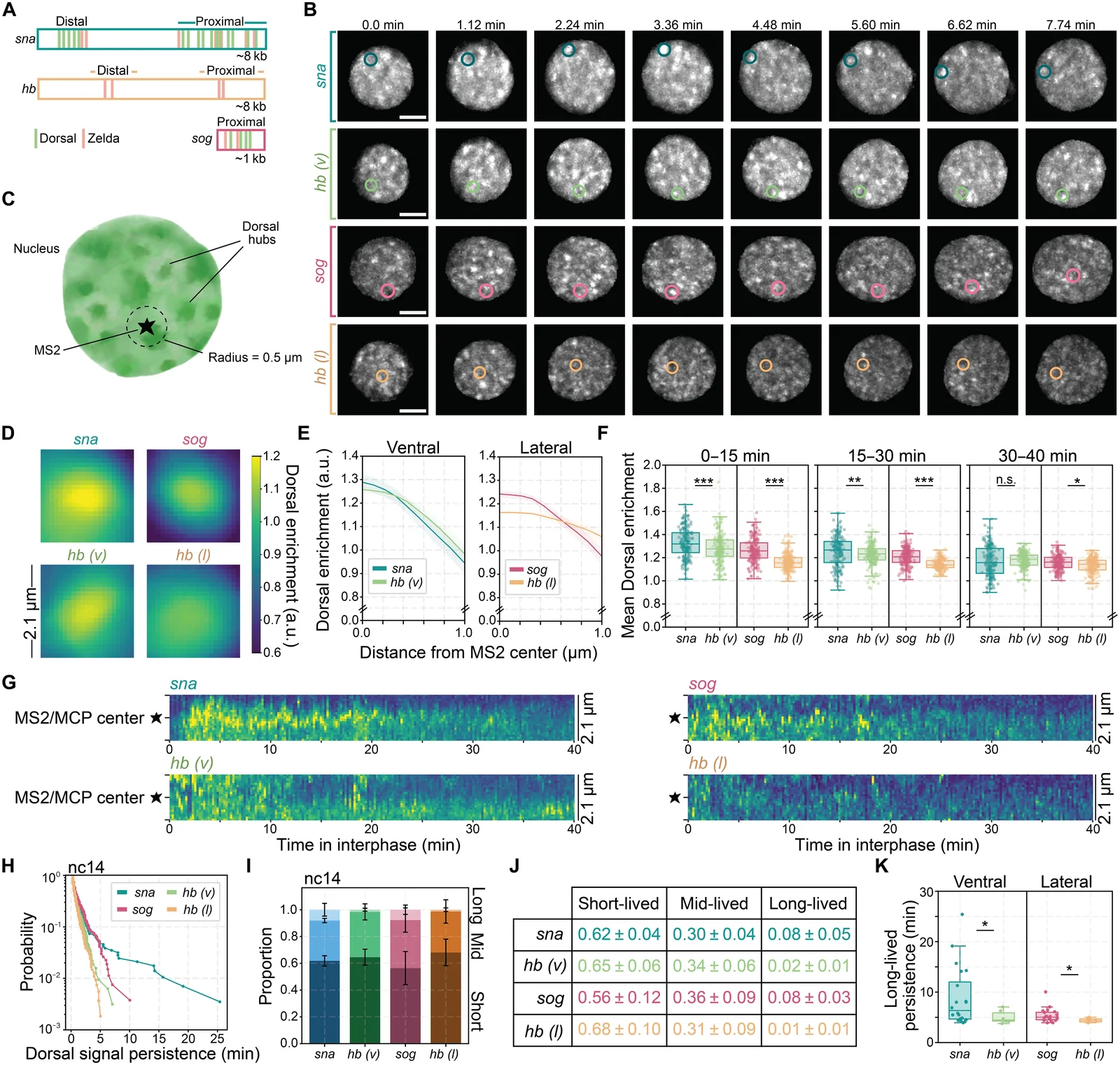
Dorsal accumulations are persistent at target genes and transient at nontargets. (**A**) Schematic of *sna*, *sog*, and *hb* enhancers showing Dorsal and Zelda (a cofactor common to all three) binding sites. Each enhancer was placed upstream of its promoter, followed by 24 MS2 sequences and yellow coding sequence (genotypes in table S1). (**B**) Representative maximum intensity projections of single nuclei with Dorsal-mNeonGreen over ∼8 min of nc14. The MS2/MCP reporter is circled in each image; cytoplasmic Dorsal signal is masked, and contrast was adjusted independently per row for visualization. Scale bars, 2 μm. (**C**) Schematic of the analysis: A nucleus with Dorsal hubs is shown, with the MS2/MCP spot marked as a star and a 0.5-μm radius sphere used to quantify Dorsal signal at the reporter. (**D**) Average Dorsal enrichment centered at the MS2/MCP site (images, 2.1 μm by 2.1 μm); color bar indicates Dorsal enrichment (intensity over nuclear mean). (**E**) Average radial profile (± SEM) of Dorsal enrichment as a function of distance from the MS2/MCP spot. (**F**) Boxplots of mean Dorsal enrichment within 0.5 μm of the MS2/MCP spot at the beginning, middle, and end of nc14 for each reporter (Mann-Whitney *U* test; *P < 0.05, **P < 0.01, and ***P < 0.001). (**G**) Representative kymographs (one nucleus per reporter) of Dorsal enrichment at the MS2/MCP spot over nc14. (**H**) Survival plots of hub persistence time within the interaction sphere for each reporter across nc14. (**I**) Stacked bar plot showing proportions of short-, mid-, and long-lived Dorsal enrichment events, classified by a three-component Gaussian mixture model, across target and nontarget loci. (**J**) Table of mean percentages and SDs for each population in (I). (**K**) Boxplots of average persistence duration within the long-lived category, with events pooled from three embryo replicates (five nuclei per embryo) per reporter (Mann-Whitney *U* test; *P < 0.05). n.s., not significant.

We built an analysis pipeline to localize and track sites of nascent transcription from the MS2/MCP signal over time and the kinetics of Dorsal interactions at these sites (Fig. 2C and movie S6). First, to quantify site-specific enrichment independently of hub segmentation, we measured the average Dorsal intensity centered on the MS2/MCP spot over time (Fig. 2, D and E). Because of the higher nuclear background in ventral nuclei, the hub enrichment is higher at *hb(v)* than *sog*. However, when comparing *hb(v)* with *sna* and *hb(l)* with *sog*, our analysis shows that Dorsal is enriched at actively transcribing target genes relative to nontarget controls. The radial profiles demonstrate that Dorsal enrichment at target loci decays substantially within ∼0.5 μm of the MS2/MCP site with *sog* enrichment approaching *hb(l)* levels at this distance (Fig. 2E). We therefore used a 0.5-μm sphere average to capture specific locus-associated signals while reducing noise and accounting for slight spatial offsets of hubs relative to the MS2/MCP spot (Fig. 2F). The mean enrichment in this sphere closely matches the trends of enrichment measured directly at the MS2/MCP center. Consistent with the nucleus wide trends (Fig. 1), Dorsal enrichment at MS2/MCP correlates strongly linearly with nuclear Dorsal concentration (fig. S3A), suggesting that hubs persistently form at target genes and the concentration of Dorsal within them scales with available nuclear Dorsal. Toward the end of nc14 as the Dorsal nuclear concentration drops, so does hub intensity, and the distinction between target and nontarget loci is diminished.

Examination of kymographs centered on MS2/MCP spots (Fig. 2G) shows that target loci exhibit extended periods of sustained enrichment compared to nontarget loci. To characterize the temporal stability of hub presence at a locus, we defined a “persistent enrichment event” as a continuous time interval when the mean Dorsal intensity within 0.5 μm of the center of the MS2/MCP spot exceeds the nuclear mean intensity for at least three consecutive frames. We observed a wide distribution of persistence times at each reporter locus. Survival probability analysis of persistent enrichment times shows a significantly increased long-lived population (i.e., stable hub formation) at target loci versus nontargets (Fig. 2H). To further characterize persistence, we fit the distribution enrichment-segment durations to a three-component Gaussian mixture model (fig. S3B) and classified segments as short-, mid-, and long-lived. Both *sna* and *sog* exhibit rare but pronounced long-lived enrichment segments (∼8% of events in nc14) (Fig. 2, I and J, and fig. S3C), consistent with prior literature reporting infrequent but stable hub associations to genomic sites (*16*, *22*). However, the long-lived population is significantly more persistent at *sna* than at *sog* suggesting site-specific or nuclear concentration–dependent effects (Fig. 2K).

Since the experiments described above were performed using a transgenic reporter inserted in the same genomic location, to assess differences at endogenous gene loci, we repeated these experiments with the same imaging conditions but now using MS2 knock-in *sna* and *sog* fly lines containing 24 MS2 stem loops placed in the 3′ or 5′ untranslated region at each endogenous gene locus (fig. S3, D to K, and movie S7) (*44*). Because of *sog* being on the X chromosome, we solely imaged female embryos, while *sna* embryos were not separated by sex for analysis. In these endogenous knock-ins, relative enrichment and elevated persistence were preserved. Notably, endogenous *sna* exhibited a markedly higher proportion of long-lived enrichment segments (∼40%) (fig. S3K). Absolute enrichment values and persistence differed slightly from transgenic reporters. These differences could be due to several factors. For instance, in the case of *sog*, the endogenous locus contains additional enhancers, and for both genes, the chromatin context is different from that of the transgenic reporters inserted in a gene desert. In addition, these genes have different nuclear positions [e.g., *sna* tends to be apically positioned (*45*)] that may affect the ability to call specific hub localization due to variation in surrounding genomic site density. For example, lower surrounding site density could lead to enhanced hub detection near the nuclear surface thus enhancing enrichment and persistence measurements. Despite these differing values, quantifying enrichment and persistence in the endogenous condition still leads to the conclusion that Dorsal hub properties are specific and tunable at different target loci. However, because of the different concentrations within nuclei that express *sog* and *sna*, absolute persistence values cannot be directly compared between these loci; therefore, we next leveraged the natural concentration gradient within the *sog* expression domain to isolate the effects of nucleoplasmic background.

To assess the effects of Dorsal nuclear concentration, we imaged the *sog* reporter near the dorsal-lateral transition point where Dorsal concentration is relatively low [as in fig. S1 (A and B)] and compared them to more ventrally positioned nuclei that remain within the *sog* domain but have higher nuclear concentrations. We refer to these populations as *sog^lateral^* and *sog^medial^*, respectively (fig. S4, A and B, and movie S8). We observed clear differences in Dorsal enrichment relative to the nuclear mean between these two populations (fig. S4, C to E). In medial nuclei where nucleoplasmic Dorsal concentration is higher, the relative enrichment at the locus appears reduced. This reduction reflects an increased background intensity, since as the nuclear background increases, the contrast between locus-associated enrichment and the surrounding nucleoplasm diminishes. Persistence analysis revealed a reduced long-lived enrichment population in *sog^medial^* nuclei compared to *sog^lateral^* nuclei (fig. S4, F to I). This reduction in persistence does not necessarily indicate reduced binding site occupancy. Previous work has shown that for another Dorsal target gene, *zen*, Dorsal maintains similar levels of occupancy in lateral and ventral regions despite changes in nuclear concentration (*46*). Therefore, the change in persistence at *sog* can be attributed to an elevated background, where enrichment segments are more likely to fall below the nuclear mean threshold, shortening the apparent persistence of above-background enrichment events. Our persistence measurements thus represent a conservative estimate of enhancer-specific differences in hub stability.

Together, these data indicate that Dorsal enrichment and persistence are site specific. While Dorsal hubs transiently appear to associate with nontarget loci, these interactions are likely incidental, and stable hub enrichment is restricted to target genes.

### Hub enrichment and persistence do not strongly predict burst parameters

To assess whether Dorsal hub interactions are quantitatively linked to transcriptional kinetics, we analyzed the relationship between hub properties and transcriptional burst kinetics (Fig. 3A). Transcription occurs in bursts corresponding to transitions between inactive and active promoter states (*47*). We segmented MS2/MCP fluorescence traces normalized to MCP background levels into individual bursts by identifying changes in the rate of increase of MS2/MCP signal (see Materials and Methods) (Fig. 3B). For each reporter, we extracted burst parameters including amplitude (amount of transcripts), loading rate (slope of rise), total output (integrated intensity), duration of bursts, and frequency (interval between bursts) (*48*). We then quantified three hub-related features at each locus: (i) Dorsal intensity and (ii) Dorsal enrichment at the MS2/MCP site at the burst onset and (iii) the fraction of the burst duration with persistent Dorsal enrichment (Fig. 3C and fig. S5).

**Fig. 3.**
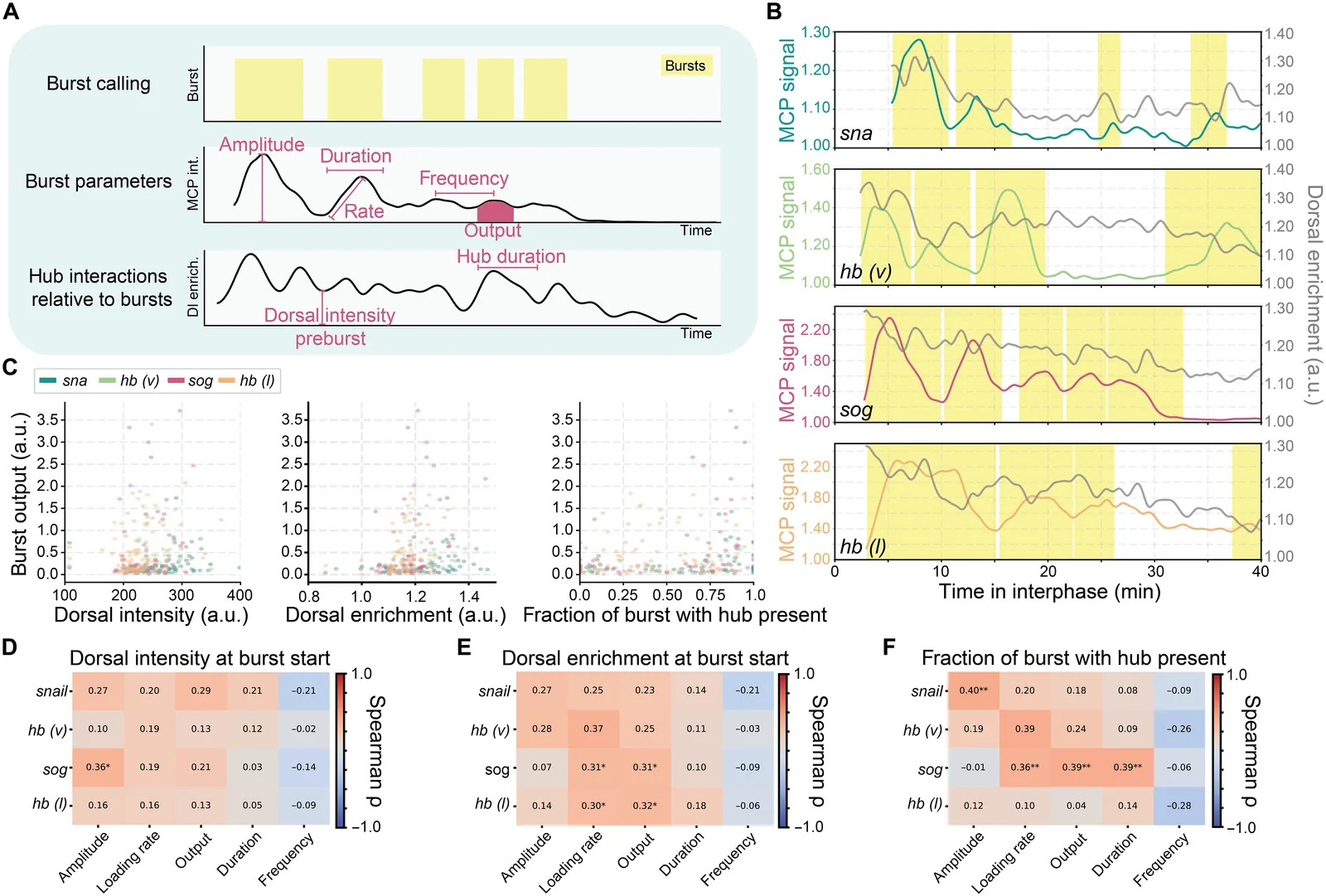
Dorsal hubs show minimal correlation with transcriptional burst kinetics. (**A**) Schematic showing the burst calling, burst parameters extracted from the MS2/MCP intensity, and the hub interaction parameters quantified from the Dorsal hub overlap and intensity. Bursts are identified in yellow. (**B**) Representative traces of MS2/MCP intensity (colored) and Dorsal intensity (gray) at each reporter gene in nc14. (**C**) Scatterplots showing burst output with the mean Dorsal intensity before the burst (left), the mean Dorsal enrichment before the burst (middle), or the fraction of time during a burst when a hub is persisting near the MS2/MCP site (right). Marker colors correspond to each reporter analyzed. (**D** to **F**) Heatmaps show the Spearman’s rank correlation coefficient (ρ) between the Dorsal intensity at burst start (D), Dorsal enrichment at burst start (E), and fraction of burst with hub present (F) for each gene and each burst parameter. The asterisks indicate *P* values: *P < 0.05 and **P < 0.01.

Across target and nontarget reporters, correlations between hub metrics and burst parameters were minimal (Fig. 3, D to F). Because the MS2/MCP signal is detectable only after transcription initiation, these analyses are restricted to periods of active transcription and do not capture hub behavior preceding burst onset. The physical position of the locus cannot be reliably tracked during extended transcriptional off-intervals. While the locus position could be interpolated between active bursts, the rapid, independent three-dimensional (3D) motion of the chromatin locus risks spatial misalignment. Such interpolation errors could lead to false positives or negatives when calling local Dorsal enrichment or tracking hub persistence. Therefore, to ensure data integrity, metrics requiring accurate off-time tracking were excluded from this analysis. It remains a distinct possibility that hub dynamics occurring before or in between a transcriptional burst are key drivers of burst parameters or that off-times or intervals between bursts could be correlated with hub properties. Nevertheless, our results indicate that while Dorsal hubs are enriched at target loci, variation in hub intensity or persistence at a given enhancer at the start of or during a transcription burst does not robustly predict burst parameters.

### Reducing Dorsal motifs within *sna* decreases hub enrichment and persistence

To test whether enhancer binding site number directly modulates Dorsal hub properties, we leveraged the dual-enhancer structure of the *sna* locus. We focused on this locus to remove the concentration effects between enhancers active in different concentration regions (like *sog* and *sna*). The full *sna* reporter contains both the proximal and distal enhancers (*42*), so we compared this construct to reporters containing only the proximal enhancer (*snaPE*) (gift from B. Lim) or only the minimal distal enhancer (*snaDE*) (*39*), progressively reducing the total number of Dorsal binding sites (Fig. 4, A and B, and movie S9). We included *hb* imaged on the ventral surface [*hb(v)*] as a negative control. Quantification of Dorsal enrichment at the MS2/MCP site revealed a reduction in enrichment from *sna* to either *snaPE* or *snaDE* (Fig. 4, C to E). Notably, enrichment of both *snaPE* and *snaDE* approached levels indistinguishable from *hb(v)*, suggesting that a minimum density of binding sites may be required to generate enrichment detectable above the nuclear background. Although absolute levels remained near background, both *sna* and *snaPE* displayed significantly higher proportions of long-lived enrichment segments and longer persistence durations compared to *snaDE* and *hb(v)* (Fig. 4, F to I). In contrast, persistence at *snaDE* was statistically indistinguishable from *hb(v)*. These results indicate that increasing Dorsal binding site number enhances both enrichment magnitude and persistence, and differences between three binding sites and the nonspecific control are indistinguishable in our assay.

**Fig. 4.**
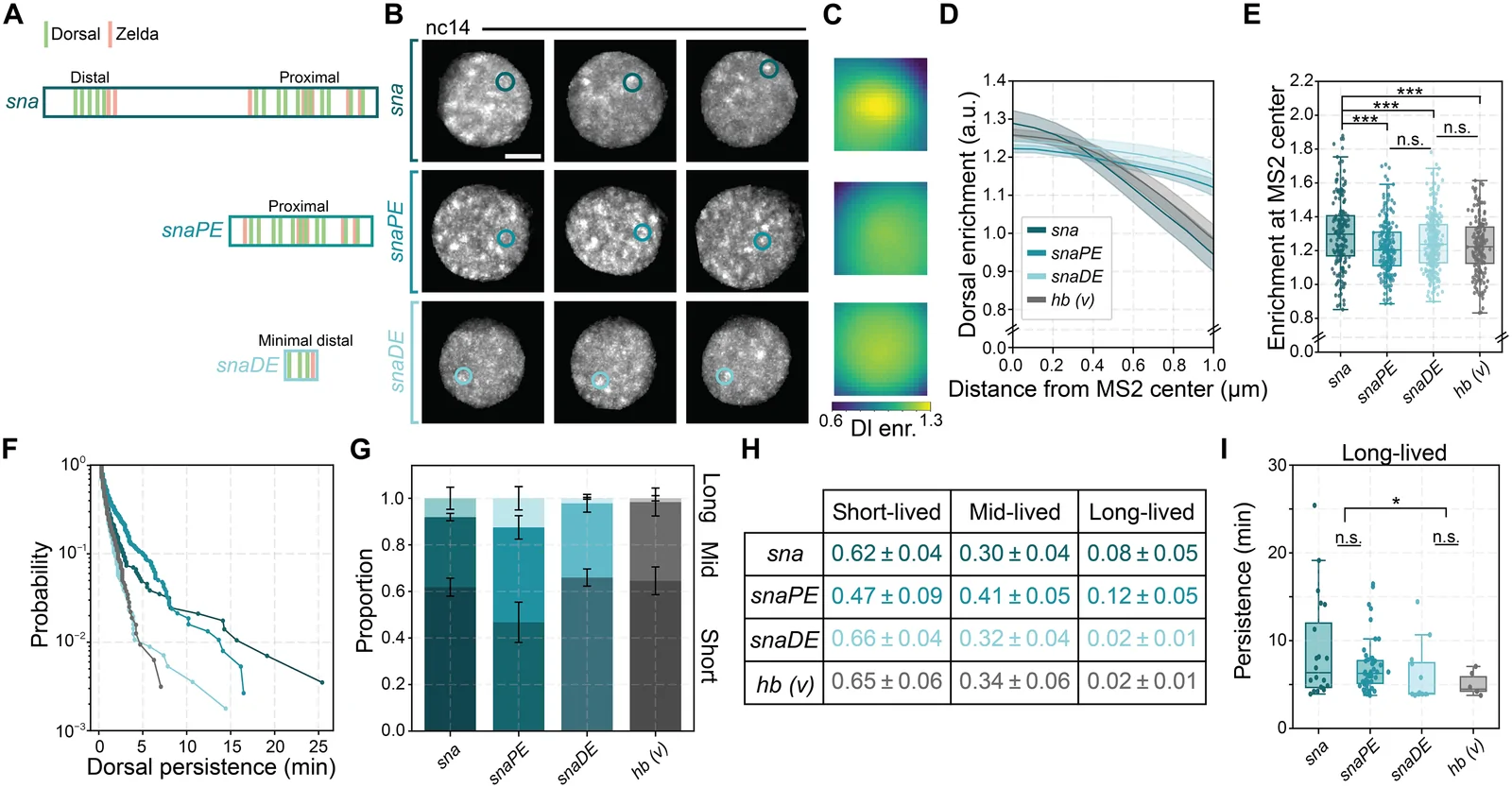
Reducing Dorsal motifs decreases hub enrichment and persistence. (**A**) Schematic of enhancers with Dorsal and Zelda binding sites for each *sna* reporter: both enhancers (*sna*, ∼16 Dorsal sites), the proximal enhancer alone (*snaPE*, ∼11 sites), or the distal enhancer alone (*snaDE*, ∼3 sites). Each enhancer was positioned upstream of its promoter, followed by 24 MS2 sequences and yellow coding sequence (genotypes in table S1). Reporters were imaged along the ventral surface in nc14. (**B**) Representative maximum intensity projections of single nuclei with Dorsal-mNeonGreen in nc14. The MS2/MCP spot is circled in each image; cytoplasmic Dorsal signal is masked, and contrast was adjusted independently per row for visualization. Scale bar, 2 μm. (**C**) Average Dorsal enrichment centered at the MS2/MCP site (images, 2.1 μm by 2.1 μm); color bar indicates Dorsal enrichment (intensity over nuclear mean). (**D**) Average radial profile (± SEM) of Dorsal enrichment as a function of distance from the MS2/MCP spot. (**E**) Boxplots of Dorsal enrichment at the MS2/MCP spot center [radius = 0.0 μm in (D); Mann-Whitney *U* test, n.s. = non-significant (*P* > 0.05) and ***P < 0.001). (**F**) Survival plots of hub persistence time within the interaction sphere for each reporter across nc14. (**G**) Proportions of long-, mid-, and short-lived hub formation events per bin, with error bars showing SD among embryo replicates. (**H**) Table of mean and SD for short-, mid-, and long-lived hub persistence proportions from (G). (**I**) Boxplots of average duration of consistent hub interactions within the long-lived population. Persistence events plotted: *sna*, 18 nuclei per three embryos; *snaPE*, 22 nuclei per three embryos; *snaDE*, 25 nuclei per four embryos; *hb(v)*, 18 nuclei per four embryos (Mann-Whitney *U* test; n.s. = non-significant (*P* > 0.05) and *P < 0.05).

To further probe the sensitivity limits of our detection, we imaged additional reporters in which one or two Dorsal binding sites were removed from the distal enhancer (*49*) (*snaDE-1xdl* and *snaDE-2xdl*; fig. S6, A and B, and movie S10). Across these constructs, neither enrichment nor persistence differed significantly from the unmodified *snaDE* reporter or from *hb(v)* (fig. S6, C to H). These data indicate that further reductions in binding site number within the distal enhancer fall below the threshold required for detectable changes in hub properties in our assay.

We next tested whether altering non-Dorsal binding sites within the enhancer influences hub behavior. Zelda is a pioneer TF known to regulate early embryonic genes and to form its own nuclear hubs (*9*, *39*). We compared the distal enhancer reporter to a modified reporter containing an additional Zelda binding site added 5′ (*snaDE + 1zld*) (Fig. 5, A and B, and movie S11) (*39*). Unexpectedly, addition of a single Zelda binding site resulted in a small but significant decrease in Dorsal enrichment at the MS2/MCP site (Fig. 5, D, F, and H) without altering Dorsal persistence (Fig. 5, J and L, and fig. S7, A to D). We then examined Zelda localization at these reporters using an endogenously tagged mNeonGreen-Zelda line (Fig. 5C and movie S12) (*9*). Zelda enrichment increases significantly at *snaDE + 1zld* relative to *snaDE* (Fig. 5, E, G, and I) although Zelda persistence remains unchanged (Fig. 5, K and M, and fig. S7, C and D). While Zelda famously acts as a pioneer factor to facilitate Dorsal recruitment globally (*50*), our data suggest that this relationship is highly sensitive to local cis-regulatory architecture. A spacing of 32 bp places the two TFs in a range where steric hindrance or unfavorable orientation could disrupt cooperative assembly, resulting instead in localized spatial competition. Consequently, instead of acting cooperatively, the binding of Zelda at this specific position may physically constrain local Dorsal binding, manifesting as the modest decrease in concurrent Dorsal enrichment (Fig. 5F).

**Fig. 5.**
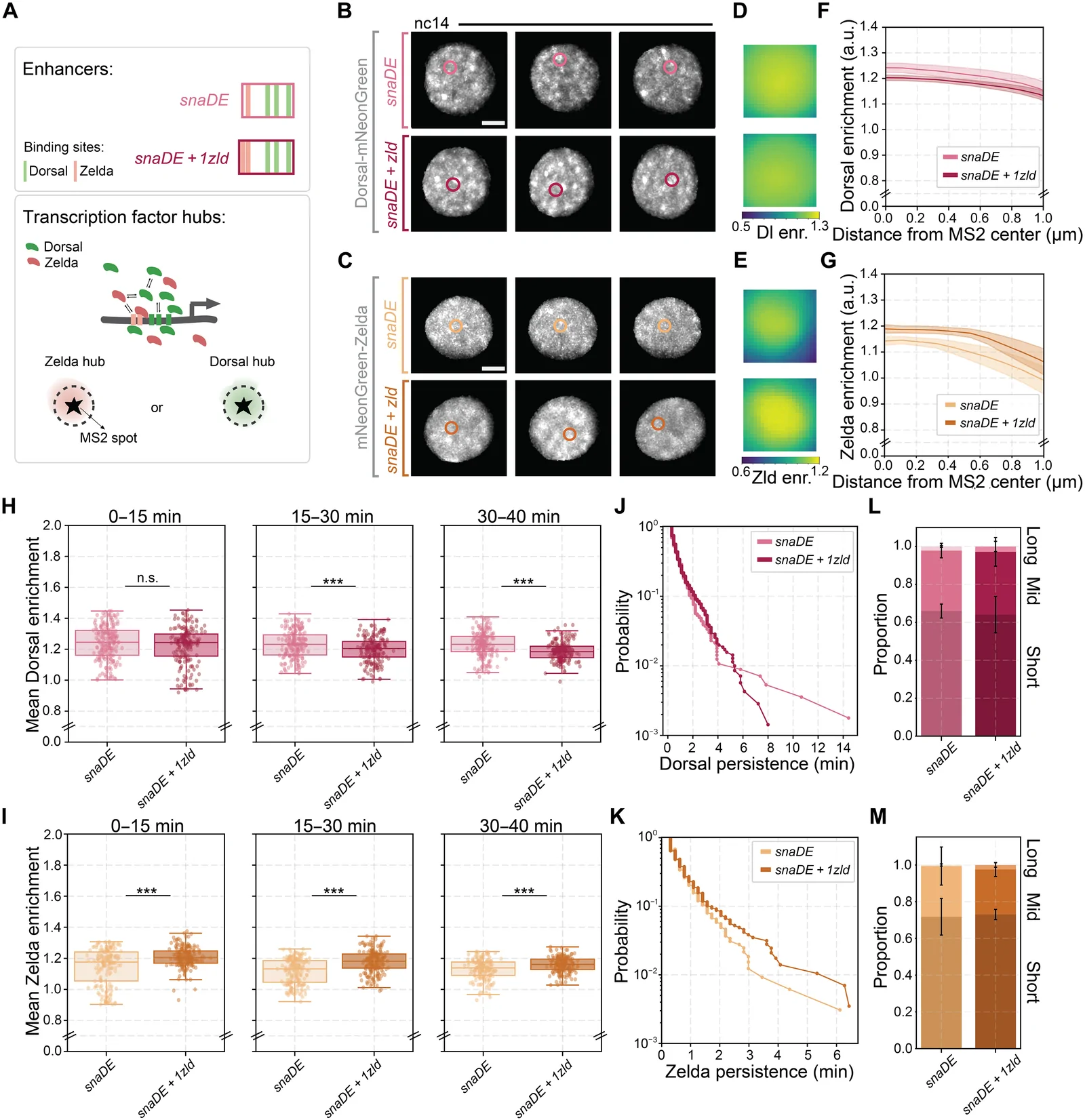
Adding a single binding site for Zelda increases Zelda enrichment at the *sna* distal enhancer. (**A**) Schematic of Dorsal and Zelda binding sites for the *sna* distal enhancer reporter without (*snaDE*) or with (*snaDE + 1zld*) an added Zelda site. Each enhancer was positioned upstream of its promoter, followed by 24 MS2 stem loops and yellow coding sequence (genotypes in table S1). Reporters were imaged at the ventral surface in nc14, with either Dorsal-mNeonGreen or mNeonGreen-Zelda to visualize hubs formed by each TF. (**B** and **C**) Representative maximum intensity projections of single nuclei with Dorsal-mNeonGreen (B) or mNeonGreen-Zelda (C) in nc14. The MS2/MCP spot is circled in each image; cytoplasmic signal is masked, and contrast was adjusted independently per row for visualization. Scale bars, 2 μm. (**D** and **E**) Average Dorsal (D) or Zelda (E) enrichment centered at the MS2/MCP site (images, 2.1 μm by 2.1 μm); color bar indicates local intensity over nuclear mean. (**F** and **G**) Average radial profile (± SEM) of Dorsal (F) or Zelda (G) enrichment as a function of distance from the MS2/MCP spot. (**H** and **I**) Boxplots of mean Dorsal (H) or Zelda (I) intensity at the beginning, middle, and end of nc14 for each gene (Mann-Whitney *U* test; ***P < 0.001). (**J** and **K**) Survival plots of Dorsal (J) or Zelda (K) hub persistence within 0.5 μm of the MS2/MCP center across nc14. (**L** and **M**) Gaussian mixture model categorization of Dorsal (L) or Zelda (M) hub persistence events into short-, mid-, and long-lived bins, with error bars showing SD among embryo replicates. *N* = *snaDE* (Dorsal), 25 nuclei per four embryos; *snaDE +1zld* (Dorsal), 36 nuclei per four embryos; *snaDE* (Zelda), 24 nuclei per three embryos; *snaDE +1zld* (Zelda), 19 nuclei per three embryos.

Together, these results demonstrate that enhancer binding site composition tunes TF enrichment and persistence at actively transcribing reporter sites. Dorsal hub enrichment and persistence decrease when lowering binding site number, but these measurements are constrained by detection limits imposed by the nuclear background. Moreover, addition of a single Zelda binding site is sufficient to measurably increase Zelda enrichment while modestly altering Dorsal enrichment, suggesting that enhancer composition can differentially shape factor-specific hub properties.

### Enhancer-dependent Dorsal hub properties are dictated by molecular binding kinetics

We hypothesized that if Dorsal hub properties simply reflect binding kinetics at enhancer-encoded binding sites, then a kinetic binding model should be sufficient to recapitulate our experimental results on hub enrichment and persistence. To test this, we incorporated SMT-derived kinetic parameters into a quantitative model of TF binding (*51*). To parameterize the simulations, we performed SMT experiments in live Drosophila embryos as previously shown (*8*, *9*, *20*, *21*, *41*, *52*). We used a fly line containing endogenously tagged Dorsal-mEos4a to enable sparse photoconversion for single-molecule detection at both ventral and lateral surfaces of the embryo (*41*). Using long exposure times (500 ms) to blur out the unbound population of molecules and reduce the effects of photobleaching, we measured the residence time of Dorsal to be ∼4 s in both regions (fig. S8, A and B). Using short exposure times (10 ms) to track both bound and unbound molecules, we estimated diffusion coefficients and the immobile and free fractions (*53*) and found them to be similar (∼58%) in both ventral and lateral nuclei (fig. S8, C and D).

Using our SMT data (fig. S8), we simulated the diffusion and binding kinetics of Dorsal molecules in nc14 nuclei. We inferred the number of Dorsal binding sites (*50*) and Dorsal concentrations (*54*) from previously published data (for a full list of parameters, see table S2). We simulated diffraction-limited microscope images by convolving the simulated molecule positions with the microscope’s point spread function (PSF), mimicking the signal recorded by our camera sensor. These images were generated in 3D to obtain z-stacks over time, taking into account variables such as slice spacing and exposure time, to match our experimental data. We inserted binding site arrays to match the *sna* (Fig. 6) and *sog* enhancers (fig. S9, A to G) we examined experimentally. Using the same analysis pipelines we applied to our experimental data, we characterized hub enrichment and persistence in our simulated data.

**Fig. 6.**
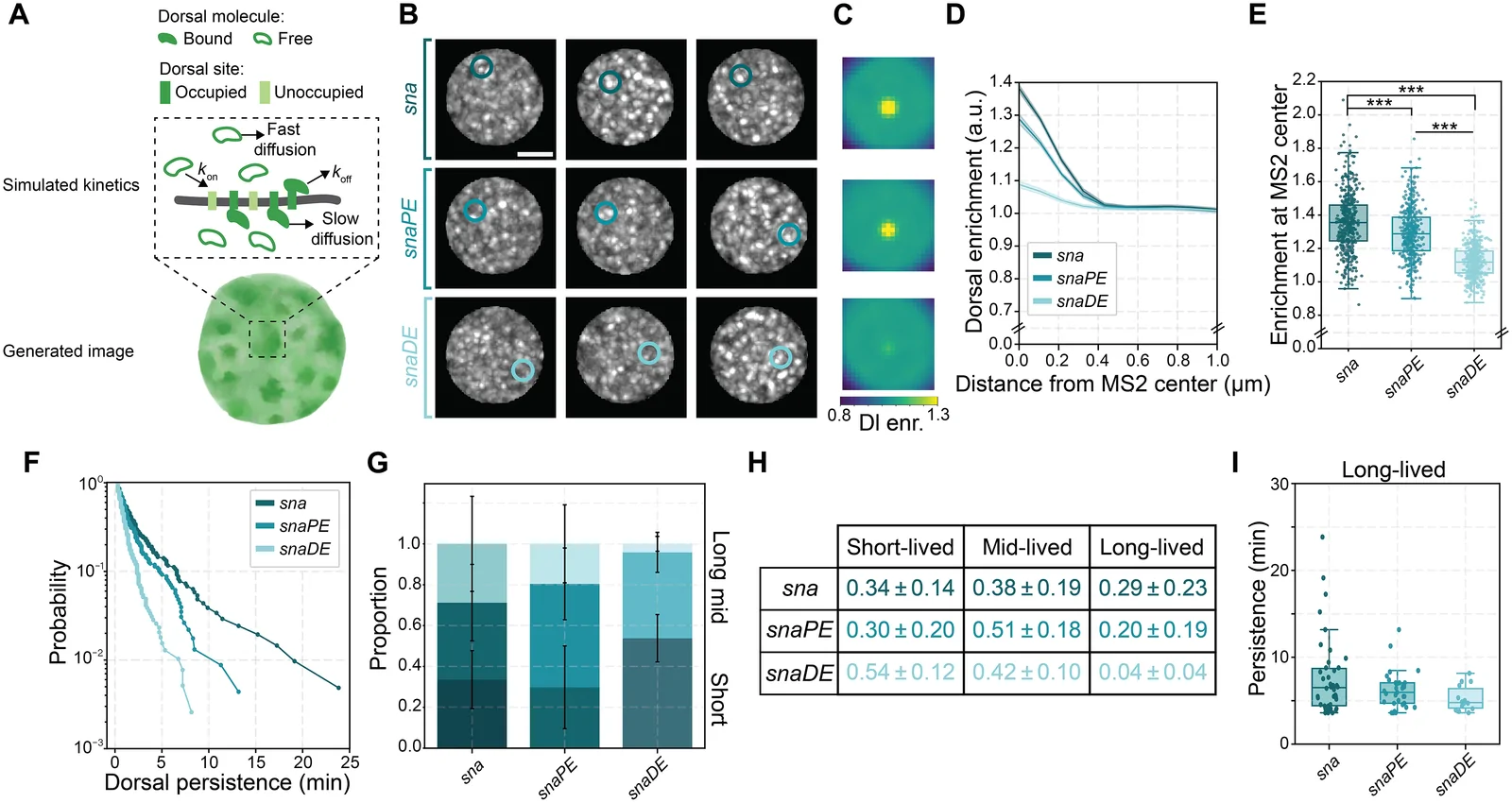
Molecular binding kinetic simulations recapitulate hub enrichment and persistence. (**A**) Schematic showing the generation of synthetic nucleus images by modeling Dorsal single-molecule kinetics and binding (*k*_on_ = binding probability; *k*_off_ = disassociation rate). (**B**) Representative maximum intensity projections of simulated single nuclei with Dorsal signal in gray. The binding site cluster is circled in each image. Contrast is scaled equally for all conditions. Scale bar, 2 μm. (**C**) Images show simulated average Dorsal enrichment centered at the binding site clusters. Images are 2.1 μm by 2.1 μm. The color bar on the bottom shows the level of Dorsal enrichment in the image (Dorsal intensity over nuclear mean intensity). (**D**) Average radial profile with SE centered at the binding site cluster shows Dorsal enrichment as a function of distance from spot center. (**E**) Boxplots showing the distribution of the Dorsal enrichment at the center of the binding site cluster (radius = 0.0 μm). A Mann-Whitney *U* test was performed to determine significance, and the following *P* values were used: ***P < 0.001. (**F**) Survival plots showing the length of time any hub is consistently present within the interaction sphere for each reporter. (**G**) The proportion of long-, mid-, and short-lived persistent hub formation in each bin is shown with error bars designating SD among embryo replicates. (**H**) The proportion of short-, mid-, and long-lived hub persistence events is listed with their mean and SD in the table. (**I**) Box plots showing the average duration of consistent hub interactions within the long-lived populations. Each persistence event from the following nuclei is plotted. *N* = 15 nuclei per simulated enhancer. A Mann-Whitney *U* test was performed to determine significance, and no significant differences were found (*P* > 0.05).

We found that our model is able to recapitulate the differences we see in hub properties at different *sna* enhancers (full, proximal, or distal) (Fig. 6). Hub enrichment scales with the number of Dorsal binding sites as in experimental data (Fig. 6, C to E); however, the enrichment magnitude is increased relative to our real data. This increase is likely due to two factors: lower background noise in the simulated images and spread of enrichment signal in our experimental data due to our reliance on using the MS2/MCP spot (i.e., the distance of the MCP-mCherry signal plus the stem loops from the enhancer) for estimating the position of the binding sites as opposed to using the precise position of binding sites in our simulations. The persistence of Dorsal signals at different reporters also follows the same trend as our experimental data, with persistence scaling with the number of binding sites (Fig. 6, F to I).

Notably, our simulations incorporate cooperativity by using a Hill-like coefficient (*n* = 100) (*55*, *56*) which dynamically scales the binding probability rather than imposing an equilibrium sigmoidal curve (see Materials and Methods). We repeated these simulations with Hill-like coefficients of 2 and 4, as reported for NF-κB (Dorsal’s mammalian homolog) (*57*) which yielded qualitatively similar results (fig. S10, A to H). Lowering the Hill-like coefficient decreases overall enrichment and persistence yet more clearly exposes binding site number scaling. Completely removing cooperativity fails to scale Dorsal persistence with site number (fig. S10, I to V).

In addition, we recapitulated the hub properties at *sog^medial^* and *sog^lateral^* and found that the increased concentration in the “medial” embryonic region did lead to lower persistence consistent with our data (fig. S9, A to G). Furthermore, we used our simulation framework to illustrate the technical limits of hub detection by evaluating how background noise affects our ability to resolve weak enrichment of *snaDE* and our negative control *hb* (fig. S9, H to N). By decreasing the simulated background noise parameter fivefold, a clear difference in enrichment and persistence emerged between *hb^low^* and *snaDE^low^* that was previously obscured by background fluctuations. This indicates that low occupancy or transient hubs at weaker enhancers are not entirely absent but rather fall just below the detection threshold of our standard imaging pipeline. Therefore, our simulations demonstrate that molecular binding kinetics and binding site number are sufficient to explain enhancer-specific hub properties.

## DISCUSSION

Our findings demonstrate that TF hubs are not homogeneous entities but instead reflect binding kinetics at specific enhancer sequences. We show that across synthetic and endogenous reporters, Dorsal hub persistence and enrichment are enhancer specific. These properties scale with the number of binding sites and nuclear concentration but only weakly correlate with transcriptional burst kinetics (Fig. 7). These observations suggest that hubs reflect the kinetics of TF binding at enhancers rather than constituting an independent regulatory layer that determines instantaneous transcriptional output.

**Fig. 7.**
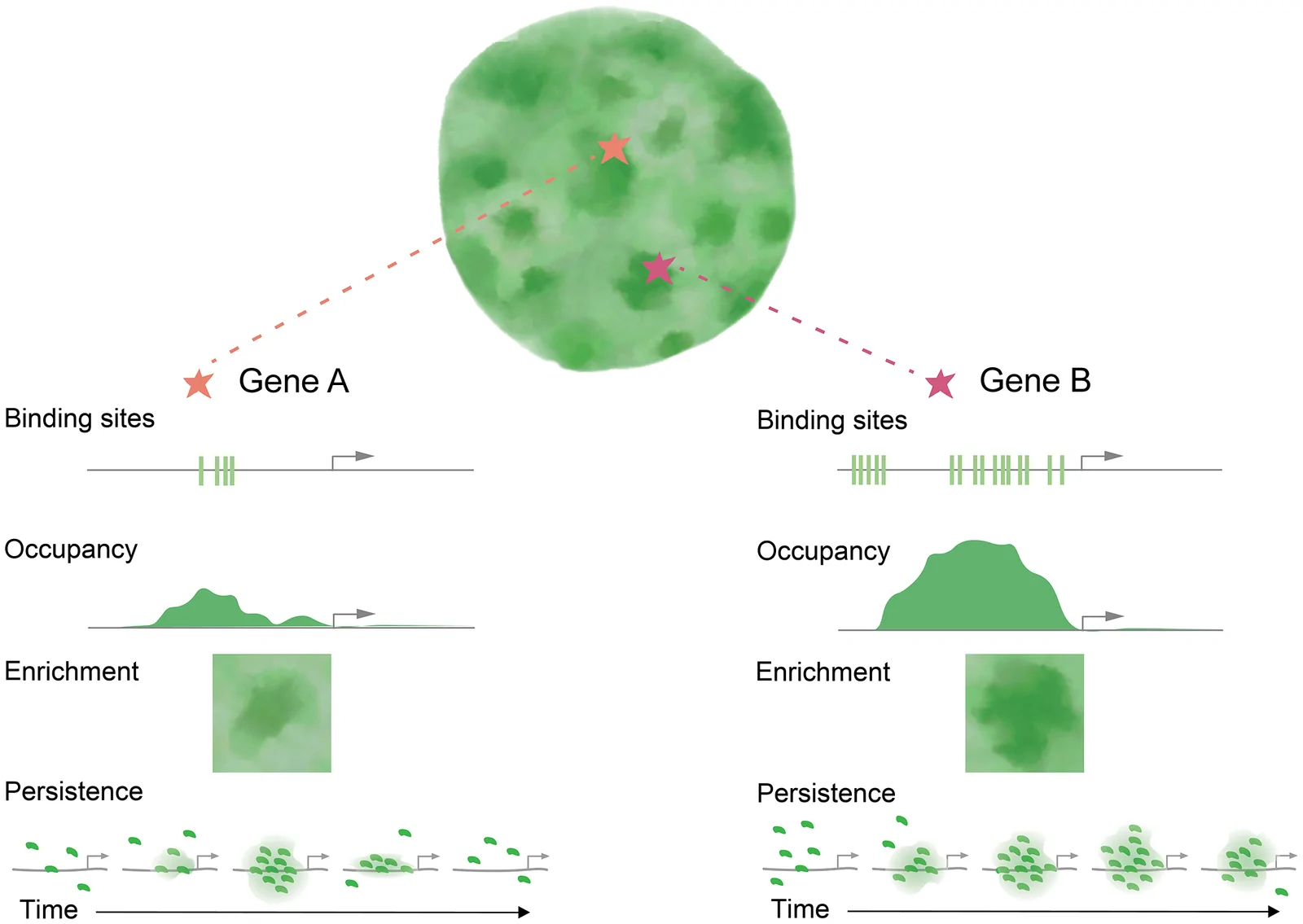
TF hub properties reflect enhancer binding site number. Binding sites at cis*-*regulatory regions mediate changes in Dorsal gene occupancy which is reflected in hub properties apparent at the locus. As a result, different target sites with lower (e.g. Gene A) or higher (e.g. Gene B) number of binding sites have differing enrichment and persistence of Dorsal hubs.

We found that the nuclear density of Dorsal hubs does not depend on nuclear concentration, while hub enrichment does. The decoupling of density from concentration is inconsistent with models where hubs arise from concentration threshold–dependent assembly of condensates. Although Dorsal clustering has been suggested to be concentration dependent and arising from thermodynamic phase separation (*40*), our results indicate that hub density is not dictated by bulk nuclear levels. Instead, nuclear concentration appears to tune factor enrichment at existing genomic binding sites rather than creating new assemblies. This is further supported by our computational modeling which recapitulates hub properties such as enrichment and persistence through binding kinetics alone.

The cooperativity dependence required to reproduce hub persistence indicates that independent Dorsal binding is insufficient and that local cooperative effects are important. Dorsal has been modeled with high Hill coefficients (∼100) to account for switch-like gene expression patterns (*55*, *56*). This parameter could reflect local effects, where initial binding increases the probability that subsequent molecules rebind locally before escaping or known cooperativity with cofactors such as Zelda, Snail, and Twist (*58*–*61*). Notably, testing moderate exponents yields qualitatively similar clustering behavior (fig. S10, A to H). Lowering the exponent decreases absolute enrichment and persistence yet more clearly exposes binding site number scaling by removing the compression caused by extreme switch-like filtering. The fact that minimal cooperativity is sufficient to produce persistent hubs demonstrates that extreme, switch-like behavior is not strictly required. Crucially, whether driven by intrinsic cooperativity, local kinetic shifts, or cofactor interactions, these mechanisms remain rooted in sequence-encoded binding kinetics. Thus, phase separation–like mechanisms are not necessary to explain these observations.

By using reporters integrated in a common genomic docking site, we show that increasing Dorsal binding site density increases enrichment and persistence. Conversely, reducing binding sites progressively diminishes detectable enrichment and long-lived interactions, eventually reaching levels indistinguishable from nonspecific control sites. These effects extend beyond Dorsal itself. The addition of a single Zelda binding site measurably increases Zelda enrichment while modestly reducing Dorsal enrichment, highlighting that enhancer composition differentially tunes factor-specific hub properties. Cis-regulatory element control of TF hubs is not completely unprecedented. Prior work demonstrated that removing Zelda binding sites at *sog* reduced both Dorsal clustering and transcription, particularly in lateral regions where Dorsal concentration is lower (*32*). Other work also demonstrated that high-affinity Bicoid binding sites did not result in increased Bicoid clustering intensity, suggesting that clustering is not simply dictated by affinity (*38*). However, this prior study did not account for differences in the number of binding sites or hub persistence, which may be a crucial distinguishing factor, akin to the differences observed between *sna* and *sog* in our data. Overall, our data suggest that enhancer grammar may play a key role in determining hub properties and function. A key implication of our results is that persistence and enrichment can be interpreted as real-time occupancy readouts. Genomic approaches such as ChIP-seq have shown that the motif number and affinity correlate with TF occupancy. Here, our time-averaged enrichment measurements correlate with binding site number across genes in vivo, suggesting that hub enrichment reflects binding kinetics at enhancer sites.

Our data reveal that hub properties are only weakly predictive of transcription burst kinetics. In line with our data, previous studies on Gal4 clusters found no difference in burst size when the clusters were positioned closer to their target site and in vivo SMT of Gal4 at its target site suggests that burst duration and size are primarily determined by TF dwell time and binding affinity (*35*). Although stable enrichment is more prevalent at target genes, variation in enrichment or persistence during active transcription does not strongly scale with burst kinetics. However, because our analysis is restricted to active phases of the transcription cycle, we cannot rule out the possibility that hub properties influence parameters during inactive periods such as the duration between bursts. Furthermore, our current framework does not evaluate the first passage time of RNA polymerase II (Pol II) following mitosis, leaving open the possibility that hub properties before the initial transcription burst of nc14 are linked to downstream regulatory effects. Our data nonetheless suggest that while binding site composition tunes occupancy, transcriptional bursting is likely influenced by steps downstream of TF recruitment.

As has been shown previously, the presence of a TF hub is not sufficient for transcription initiation (*33*). We previously examined the effect of enhancer placement relative to the promoter (*62*). Downstream placement of the enhancer significantly dampened transcriptional output, yet we detected no change in local Dorsal enrichment between upstream and downstream configurations. This dissociation between enrichment and transcription further supports the conclusion that hub formation and enrichment are dictated by binding site composition rather than act as transcriptional modulators at a given enhancer. While our initial observations here suggest that hub properties at burst onset are not strong predictors of burst parameters at a single locus, whether these features are directly linked remains an open question. Our work does not rule out hub contributions before activation or between bursts as previously reported (*22*, *33*, *63*). In addition, recent work emphasizes that cooperative TF binding and exchange can stabilize high-occupancy states that underlie transcriptional bursting (*34*, *64*). We similarly find in our modeling that cooperativity is required to reproduce the measured hub properties. Still, the differences in burst kinetics and transcriptional output between genes are likely dependent on the level of TF occupancy which is reflected in the larger differences in hub properties between reporters.

Our findings also highlight a critical practical consideration: Hub detection is inherently constrained by the nuclear background and the contrast of the imaging modality being used. As nuclear Dorsal concentration increases, the background intensity is elevated, reducing contrast between locus-associated enrichment and the surrounding nucleoplasm without necessarily altering underlying binding kinetics at a locus. This limitation in detection could easily lead to misleading conclusions that condensates or hubs are discrete regulatory structures that highly concentrate molecules. Our recent work on RNA Pol II clustering similarly demonstrates that Pol II clusters at active genes can be explained by transcription-dependent engagement and recruitment kinetics and are thus only visible at genes that sufficiently accumulate Pol II (*20*). Our current study is also limited by its focus on a single TF. Because these hubs are emergent properties driven by enhancer composition, understanding how multiple molecules work together at the locus is critical to seeing how TFs and transcriptional machinery jointly affect local occupancy. Developing multifactor models of binding kinetics to explore how TFs interact with both DNA and one another remains an important future endeavor.

Together, our results suggest the clustering of Dorsal, and other TFs, observed in vivo may be interpretable through the lens of binding kinetics and occupancy rather than phase separation. Second, they underscore the importance of cooperative binding and enhancer features in shaping apparent hub behavior. Third, they highlight how detection thresholds and nuclear background can influence conclusions about condensate formation. Last, by demonstrating that enhancer perturbations produce quantifiable changes in enrichment and persistence, our study establishes that hub properties are encoded within DNA sequence. We suggest that the many reports of TFs and regulatory proteins forming condensates or hubs through higher-order mechanisms should be examined through the lens of molecular binding kinetics. Instead, rapid search processes may facilitate a high on-rate and frequent exchange of TF molecules at their targets, leading to the appearance of persistent hubs at gene loci despite short residence times. Future work combining enhancer dissection, kinetic modeling, and hub molecular composition analysis will further clarify how generalizable this binding and search process centric framework is across TFs and developmental systems.

## MATERIALS AND METHODS

### Embryo preparation for live imaging

For each MS2 experiment, *Drosophila* embryos were generated by crossing virgin female flies heterozygous for MCP-mCherry (III) and Dorsal-mNeonGreen to males containing the MS2 reporter of interest or males containing the endogenous MS2 knock-in. The resulting embryos were collected for live imaging. Only embryos positive for all three (Dorsal-mNeonGreen, MCP-mCherry, and MS2-gene-of-interest) were imaged. In the instance of the endogenous *sog-MS2* knock-in only, since *sog* is on the X chromosome, the active MS2/MCP spots would only be visible in female embryos as the MS2 array is only passed down via the paternal X chromosome. Thus, all embryos were screened for active MS2/MCP spots in nc13. These selected females were then chosen for imaging nc14.

Details of all lines used for imaging and where they appear in this work are listed in table S1. For SMT experiments, virgin female Dorsal-mNeonGreen; MCP-mCherry flies were crossed to male Dorsal-mEos4a flies. Flies positive for all three markers were used for imaging. Dorsal-mNeonGreen intensity was further used for assessing the position of the embryos along the dorsoventral axis; MCP-mCherry was used for assessing nuclear boundaries; and Dorsal-mEos4a was sparsely activated for single-molecule detection. Embryos were collected over a 90-min window and manually dechorionated by gently rolling them on double-sided tape using a blunt needle until the chorion was removed. Dechorionated embryos were then arranged on an agar pad before being transferred onto an adhesive heptane-acrylic spot, prepared by dissolving double-sided tape in heptane on a 25-mm-diameter glass coverslip. Embryos were manually oriented on the coverslip either ventral side up (facing the objective) or gently rotated to position the dorsal-lateral transition region within the imaging plane (fig. S1, A and B).

### Annotation of TF binding sites

TF binding sites within the enhancers were identified using the FIMO (Find Individual Motif Occurrences) tool (*65*) with position weight matrices obtained from (*66*) and the JASPAR database (*67*). The threshold for a significant motif match was set to a *P* value < 10^−3^. For *snaDE*, local annotation of Dorsal and Zelda motifs was cross-referenced with established cis-regulatory mappings (*49*, *68*). While the *snaDE* sequence lacks the canonical CAGGTAG Zelda motif, it contains an AGGTAG motif near the 5′ end on the complementary strand capable of functional Zelda binding (*69*). This endogenous motif is preserved in the engineered *snaDE + 1zld* construct, placing active Zelda sites in close proximity to each other and 32 bp away from a Dorsal binding site.

### Microscope calibration

All imaging was performed on a Multimodal Optical Scope with Adaptive Imaging Correction (MOSAIC) (*70*). For volumetric experiments, the lattice light-sheet mode was used with the 488- and 589-nm laser lines. These laser beams were first expanded to a combined 2-mm diameter, passed through a half-wave plate to adjust polarization, and relayed into an acousto-optic tunable filter (AOTF) (Quanta-Tech, AA Opto Electronic) to selectively control wavelength and power modulation. The collimated beams were then expanded in one dimension using a Powell lens (Laserline Optics, Canada), and their width was adjusted and collimated using a pair of cylindrical lenses. The beam was subsequently relayed onto a grayscale spatial light modulator (Meadowlark Optics, AVR Optics) after passing through a second half-wave plate, where it was diffracted and projected onto a custom annular mask to define the minimum and maximum numerical aperture (NA) of the light-sheet while blocking undiffracted light. The light from the annulus was demagnified and relayed onto a resonant galvanometer scanner (Cambridge Technology, Novanta Photonics), conjugate to the sample plane, to mitigate shadowing artifacts and improve uniformity by introducing a slight wobble in the excitation angle. Last, the light was projected onto a pair of galvanometer scanning mirrors (Cambridge Technology, Novanta Photonics) conjugate to the pupil plane, allowing scanning of the light-sheet along the *x* and *z* optical axes before being focused onto the sample using a Thorlabs TL20X-MPL excitation objective. Fluorescence emission was collected orthogonally by a Zeiss 20×, 1.0 NA detection objective and relayed onto a deformable mirror (ALPAO) positioned in the detection path to correct optical aberrations, as previously described (*71*). The emitted signal was then split using a Semrock Di03-R561-t3-25x36 dichroic beam splitter and directed onto two sCMOS detectors (Hamamatsu ORCA Fusion). The green emission channel (for 488-nm excitation) was filtered using a Semrock FF03-525/50-25 emission filter and a Chroma ZET488NF notch filter to reject laser light, while the red emission channel (for 589-nm excitation) was filtered using a Semrock FF01-615/24 emission filter and a Chroma ZET594NF notch filter.

### Imaging conditions

All volumetric imaging used a multibessel lattice sheet with maximum NA to minimum NA ratio of 0.4/0.3 488 nm (used to excite mNeonGreen) and 589 nm (used to excite mCherry). Two color channels were acquired sequentially for a volume of 18.9-μm sampling z-planes spaced 0.3 μm apart with an exposure time of 30 ms. A laser power of 0.124 mW was used for the 488-nm laser and 1.344 mW for the 589-nm laser. Each volume was acquired 4 s after the acquisition ended of the previous stack for a total time of ∼9.56 s in between stacks. These conditions were kept constant for each imaging experiment aside from the SMT experiments.

For single-molecule imaging, a Gaussian light sheet was used on the MOSAIC. Briefly, after passing through the AOTF, the beam was expanded using a Powell lens and then relayed onto a custom mask for filtering. The filtered sheet was then projected onto a resonant galvanometer (Cambridge Technology, Novanta Photonics). Last, an excitation objective (Thorlabs, TL20X-MPL) was used to focus the light-sheet onto the sample. The detection path for the emitted light is the same as described above for the lattice light-sheet configuration. For this light path, the SLM was bypassed to maximize laser power at the excitation objective. For single-molecule imaging, the red emission channel (for 561-nm excitation) was filtered using a Semrock FF01-585/29 emission filter and a Chroma ZET561NF Notch filter. The 405-nm laser line was kept on constantly during the acquisition period for photoswitching. Data were acquired at 10- and 500-ms exposure times for the fast and slow single-molecule imaging experiments, respectively. The excitation laser power was optimized empirically for each exposure time to achieve sufficient contrast for SMT. The powers of the photoswitching laser were also optimized empirically to achieve low enough detection densities to enable tracking. For fast single-molecule imaging experiments, 8000 frames were acquired with 24.1 mW of the 560-nm laser, 123 μW of the 488-nm laser, and 7.3 μW of the photoswitching laser for a total acquisition time of 80 s. For slow single-molecule imaging experiments, 400 frames were acquired with 0.66 mW of the 560-nm laser, 123 μW of the 488-nm laser, and 0.46 μW of the photoswitching laser for a total acquisition time of 200 s. Powers were measured at the back focal plane of the excitation objective.

### Volumetric image analysis

All volumetric imaging datasets were preprocessed before downstream analysis using GPU-accelerated 3D deconvolution via CUDA (*72*). Each dataset was deconvolved using a Richardson-Lucy–based algorithm with eight iterations, using PSFs captured from bead images acquired on the day of imaging to ensure accurate correction. For visualization and rendering, we used a custom Imaris converter, leveraging fast TIFF and Zarr file readers to efficiently generate Imaris files (*73*). Following deconvolution, images underwent nuclear segmentation using a custom-trained model in Cellpose 2.0 (*74*). Ground truth training data were generated through a combination of micro-SAM (*75*) and manual curation on MCP-mCherry images. The trained Cellpose model was then applied to segment individual slices across each dataset. To improve segmentation accuracy, a postprocessing pipeline was developed to stitch segmented slices together and interpolate missing nuclear structures. We then implemented a nearest-neighbor tracking algorithm to follow nuclei across interphase in each nuclear cycle.

To quantify hub properties, we used a custom image analysis pipeline (*20*, *21*) . First, nuclear intensity was normalized to its mean value to assess local TF enrichments above background levels. Hubs were segmented by first applying a median filter to reduce noise, followed by image erosion and reconstruction to enhance feature contrast. The reconstructed image was then subtracted from the median-filtered image to generate a binary mask identifying high-density regions. To resolve closely associated hubs, local maxima peaks were identified and used as markers for watershed-based segmentation, ensuring separation of fused or amorphous structures. Last, region properties were extracted to quantify hub features, including integrated intensity, mean intensity, and size.

### MS2 tracking

After segmenting nuclei and hubs, we manually select nuclei for further analysis under the criteria of checking if anomalies are present in the segmentation and to check if the MS2 spot is closer than 0.2 μm to the edge of the nucleus which may skew analysis. We then generate 4D images of cropped single nuclei. These selected nuclei are then subjected to another segmentation regime this time to detect the MS2/MCP spot. This regime uses a median filter, difference-of-Gaussians filter, and then a percentile threshold to segment high intensity spots. The exact sigma values and percentile used were adjusted nucleus-to-nucleus to ensure the best possible MS2/MCP segmentation. Then, we identify the spot inside the nucleus with the highest intensities to be the likely MS2/MCP spot. We then used a nearest-neighbor algorithm to track the MS2/MCP spot in time while also manually correcting any tracking errors in each nucleus. Because of the bursty nature of transcription, we interpolate the MS2/MCP position in between bursts of visible MS2/MCP spots and extrapolate the MS2/MCP position 10 frames before the first appearance of the MS2/MCP signal while correcting for the motion and size of the nucleus.

We calculated enrichment (Dorsal intensity at a locus over the nuclear background) as previously described in (*76*). However, instead of solely using spot detection, we used the calculated MS2/MCP centroid positions tracked over time as the center position for our cropped images. We then quantified enrichment segments for our persistence measurements at the MS2/MCP spot by assessing the overlap between the hub labels and a 0.5-μm radius sphere centered at the MS2/MCP weighted centroid coordinates as we have done previously (*21*). We could then monitor the duration, intensity, and volume overlap at each locus regardless of transcriptional activity. Hub persistence durations were fit using a three-component Gaussian mixture model to establish short-, mid-, and long-lived populations. To rigorously determine the optimal number of populations, we evaluated model performance across a range of components: A three-component model was selected as it minimized both the Akaike information criterion and Bayesian information criterion.

### Burst analysis

To calculate the intensity at the MS2/MCP spot, we averaged the MCP intensity within 0.5-μm radius of the MS2/MCP centroid position. Since we used MCP tagged with the mCherry fluorophore, we checked for significant signs of photobleaching. Under our imaging conditions, nuclear MCP-mCherry retains ∼77% of its initial intensity by the end of the 40-min imaging window (fig. S5A). Crucially, we find that both the MS2/MCP spot and the nuclear background levels of MCP-mCherry bleach at equivalent, proportional rates. This uniform decay preserves a stable signal-to-noise ratio throughout the nuclear cycle. To minimize the technical impact of photobleaching and avoid relying on absolute intensity values, we normalize the MS2/MCP spot intensity directly to the nuclear background. Because the signal-to-noise ratio is maintained, this level of signal decay is minimal, and we consider this to be minimal bleaching allowing us to continue with our analysis.

To analyze transcriptional bursting, MS2/MCP trajectories during nc 14 were standardized to a 0- to 40-min timeline and smoothed using a 1D Gaussian filter (σ = 2 frames). The initial activation burst was defined by locating the peak within a 10-min window post-onset, with the burst end mapped to the first local minimum after the signal fell below a 50% relative half-height threshold. Subsequent bursts were identified using scipy.signal.find_peaks (minimum prominence of 0.03, minimum height of 0.05) and bounded by a localized ramp strength check requiring a consecutive positive slope of ≥0.003 MCP units per frame over ≥7 frames. For each segmented burst spanning, we extracted three parameters: (i) amplitude, defined as the peak value of the smoothed MCP normalized intensity; (ii) duration, calculated as the elapsed time of burst; and (iii) total output, calculated via trapezoidal integration of the baseline-subtracted MCP curve over the burst duration.

On the basis of the start times of these bursts, we could then assess whether a hub was present at the start of a burst, the arrival time of that hub overlapping with the MS2/MCP region, and then overlap intensity and volume. To assess whether a hub was present, we used two different metrics: We first assessed whether any hub voxels overlapped with the MS2/MCP region and then assessed whether a hub was present and was over a threshold intensity value (the nuclear mean intensity).

### Fast SMT analysis

To process the fast SMT data, we first removed the initial ∼1000 frames affected by fluorophore bleaching. Maximum projections were generated and input into the Cellpose-SAM model (*77*) to obtain nuclear masks. These masks were manually edited to ensure that localizations along the nuclear border were excluded. Localization and tracking of the single-molecule trajectories was performed using the open source software package quot (*78*). For the 10-ms data, the following key parameters on quot were used: (i) for detection method = “identity,” spot detection settings = “llr,” *k* = 1.3, *w* = 15, *t* = 18; (ii) for localization method = “ls_int_gaussian,” window size = 9, sigma = 1.0, ridge = 0.0001, maximum iterations = 10, damp = 0.3, camera gain = 1, camera background = 108; (iii) for tracking method = “euclidean,” pixel size (in micrometers) = 0.108, search radius = 0.7 μm, maximum blinking = 0 frames. We then filtered the list of tracked molecules output by quot using the nuclear masks as described earlier to ensure that we analyzed only trajectories localized in the nucleus.

To analyze the fast single-molecule trajectories to infer the diffusion kinetics, we used Spot-On (*53*). Spot-On is a publicly available package that fits a kinetic model to the jump length distribution of the trajectories and estimates the fraction bound and diffusion coefficients for either a two-state or a three-state model, correcting for the probability of molecules diffusing out of focus. Trajectories from all videos were concatenated into one file per gradient position. For the analysis, we used the following parameters altered from default

$$\begin{array}{c} \text{TimeGap}=12\\ \text{dZ}=0.700\\ \text{GapsAllowed}=0\\ \text{TimePoints}=4\\ \text{UseEntireTraj}=1\\ \text{JumpsToConsider}=0\\ \text{NumberOfStates}=2\\ \text{FitIterations}=2\\ \text{LocError}=0.030\\ \text{UseWeights}=1\\ \text{D\_Free\_2State}=\left[0.2656\;25\right]\\ \text{D\_Bound\_2State}=\left[0.0001\;0.2656\right] \end{array}$$

Single-molecule trajectories were taken from a minimum of three independent embryos for all positions and imaging conditions. Detailed trajectory number and the number of videos from which the trajectories were compiled can be found in each figure caption.

### Slow SMT analysis for residence times

To process the slow SMT data, the same methodology was followed as described above for fast SMT, with the exception of two quot parameters: *k* = 1.5 and *t* = 15.5. Residence times were calculated from single-molecule data acquired at 500-ms exposure times as described previously (*9*, *79*). Briefly, imaging at 500-ms exposure times effectively blurs out fast moving molecules, leaving the slowly diffusing and bound molecules for tracking. The longer exposure time enables lower excitation laser power, leading to less photobleaching and longer trajectories. The persistence of trajectories over time is used to calculate a survival probability, which is 1 − cumulative distribution function of trajectory lengths (in number of frames), providing an estimate of a genome average residence time. An objective threshold of 2 s (*9*) was applied to the minimum trajectory length to remove the effects of tracking errors and slowly diffusing molecules. Probabilities below 10^−3^ were not considered for fitting to avoid using the sparsely populated distribution tails.

The survival probability curves were then fit to a double exponential model of the form *F* *exp(−*k*_ns_ * *t*) + (*1* − *F*) * exp(−*k*_s_ * *t*) using the curve_fit function in Python where *k*_s_ is the slower off-rate and *k*_ns_ is the faster/nonspecific off-rate. An exponential weighting function was used to ensure proper estimation of the slower off-rate so that the fit was not dominated by the more numerous early–time point trajectories. As the inferred off-rate *k*_s_ is biased by photobleaching and chromatin motion, bias correction was performed as previously described: *k*_s,true_ = *k*_s_ − *k*_bias_, where *k*_bias_ is the slower-off rate estimate from fitting the survival probabilities from H2B-mEos4a trajectories imaged in the same conditions. The genome wide residence times were then calculated as 1*/k*_s,true_*.* Errors reported were estimated from the SDs of the fit parameters using standard propagation of error methods.

### Simulations

Simulation modeling Dorsal diffusion and binding were developed in Python. Nuclei in nc14 were modeled as spheres of 5 μm, and Dorsal molecules were initialized at random positions within the spheres. The concentration of Dorsal along the gradient was estimated using the known ventral concentration, 500 nM (*54*), and nuclear mean intensity ratios from our live imaging data. Dorsal binding sites are also distributed in clusters of varying densities. Each binding site is assigned a residence time with probabilities broadly based on the survival curves in fig. S8A, with a greater proportion of sites having shorter residence times. A three-state model was used with Dorsal molecules being either freely diffusing, specifically bound or nonspecifically bound. Free diffusion was modeled by Brownian motion with a simulation time step of 10 ms to mimic our SMT. At each time step, molecules within a 50-nm capture radius of an unoccupied binding site can bind the site with a certain probability *k*_on_, reflecting the accessibility of the site. Once bound, the molecule diffuses with the binding site, which undergoes fractional Brownian motion to mimic the subdiffusive motion of DNA (Rouse polymer model). This is done via the Davies-Harte algorithm, on top of which a Gaussian jiggle is added to represent local fluctuations. For memory efficiency, fractional Brownian motion increments are generated per display frame and then bridged together such that subsequent frames are conditioned on previous ones. After the residence time of the binding site, the molecule unbinds and returns to the freely diffusing state.

To account for local cooperative interactions without enforcing an equilibrium sigmoidal response, we modeled cooperativity as a dynamic, occupancy-dependent increase in binding probability (*k*_on_). When a site in a cluster is evaluated for binding, the effective binding probability *k*′_on_ is scaled according to current local occupancy: *k*′_on_ *= k*_on_ * [1 + (*S*_coop_ * *N*_occupied_)*^n^*] where *k*_on_ is the baseline binding probability, *S*_coop_ is the cooperativity strength parameter (set to 1.0), *N*_occupied_ is the number of currently occupied binding sites within the cluster of binding sites, and *n* is a cooperative scaling exponent. Rather than a classical equilibrium Hill coefficient, which dictates fractional occupancy output, the exponent *n* acts as a kinetic modulation parameter that increases the *k*_on_ as bound molecules accumulate. In short, when cooperativity is enabled, the presence of a bound molecule within a cluster of binding sites increases the *k*_on_ for subsequent molecules based on n which we term a “Hill-like” coefficient.

Simulated z-stacks were recorded at intervals of 10 s with a camera exposure time of 30 ms. The molecules intensities at each simulated step within the exposure time are added together and projected on a 3D voxel grid, with an *xy* spacing of 108 nm (corresponding to the pixel size on the MOSAIC camera) and a *z* spacing of 300 nm (corresponding to the width of our z-stacks). Each molecule is convolved with a PSF, an anisotropic Gaussian with lateral full width at half maximum (FWHM) of 250 nm and axial FWHM of 600 nm, the estimated postdeconvolution blur obtained by deconvolving a fiducial image from the MOSAIC with a theoretical PSF. We additionally incorporated the background noise from the camera (100 ± 5 a.u.). More about these simulations and a user-friendly interactive tool to experiment on other TFs can be found here (*51*).

## Supplementary Materials

### The PDF file includes:

Figs. S1 to S10 Tables S1 and S2 Legends for movies S1 to S12 References
