# Supplementary material for: Enhancer binding kinetics explain transcription factor hub formation
Source: Sci Adv. 2026 Oct 9;12(41):eaeh1086. doi: 10.1126/sciadv.aeh1086 (PMC13651869; doi:10.1126/sciadv.aeh1086)
Supplement: Supplementary file 1 [file sciadv.aeh1086_sm.pdf]

Science Advances
AAAS

Supplementary Materials for

Enhancer binding kinetics explain transcription factor hub formation

Samantha Fallacaro *et al.*

Corresponding author: Mustafa Mir, mustafa.mir@pennmedicine.upenn.edu

Sci. Adv. **12**, eaeh1086 (2026)
DOI: 10.1126/sciadv.aeh1086

The PDF file includes:

Figs. S1 to S10
Tables S1 and S2
Legends for movies S1 to S12
References

Other Supplementary Material for this manuscript includes the following:

Movies S1 to S12

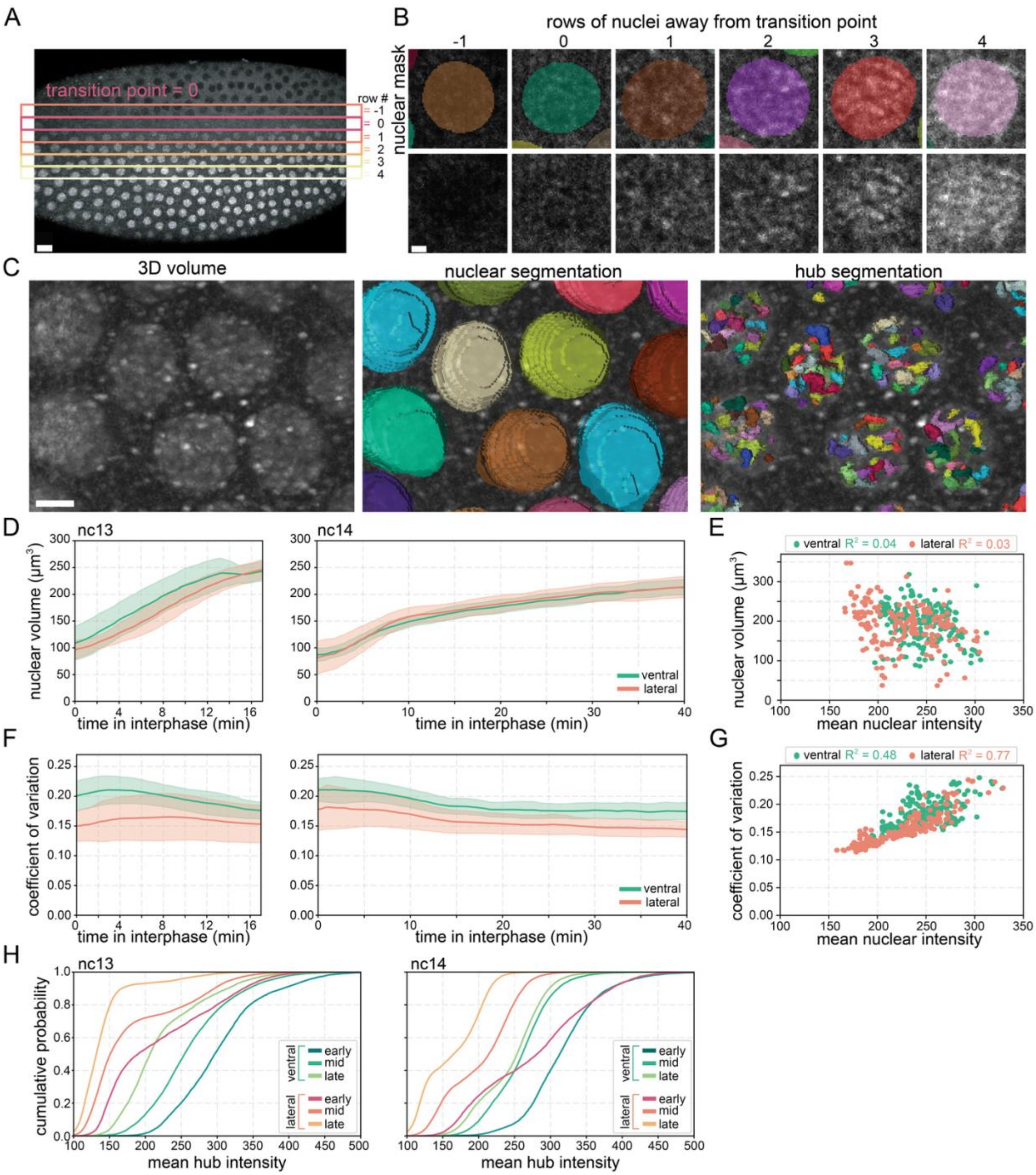

Fig. S1. Nuclear segmentation and hub detection across the dorsoventral axis.

(A, B) Images showing where nuclei were selected at the lateral surface within a max projection of a full field of view **(A)** and for a single slice for each nucleus. **(B)** Nuclei labelled as lateral were imaged within five nuclear rows of the transition point (row 0 of nuclei where the cytoplasm and nuclear signals are indistinguishable). Each row has a varying nuclear to cytoplasmic ratio of signal, yet all have hubs within the nucleus. Scale bar in B is 15 microns and 1 micron in C. **(C)** Images showing results of nuclear and hub segmentation. Left image shows a

representative 3D rendering of Dorsal-mNeonGreen in ventral nuclei. Scale bar is 2 microns. Center image shows a 3D rendering of nuclear segmentation results. Right image shows segmentation of Dorsal hubs. **(D)** Average nuclear volume in ventral (green) or lateral (orange) nuclei. Shading shows standard deviation between embryo replicates. N = 5 nuclei per embryo, 3 embryo replicates for each surface. **(E)** Scatter plot showing nuclear volume and mean nuclear intensity. R^2 is Pearson's correlation. **(F)** Coefficient of variation per nucleus in ventral (green) or lateral (orange) nuclei. **(G)** Scatter plot showing coefficient of variation and mean nuclear intensity. R^2 is Pearson's correlation. **(H)** Cumulative probability distributions of mean hub intensity for early, mid, and late nc13 (left) and nc14 (right).

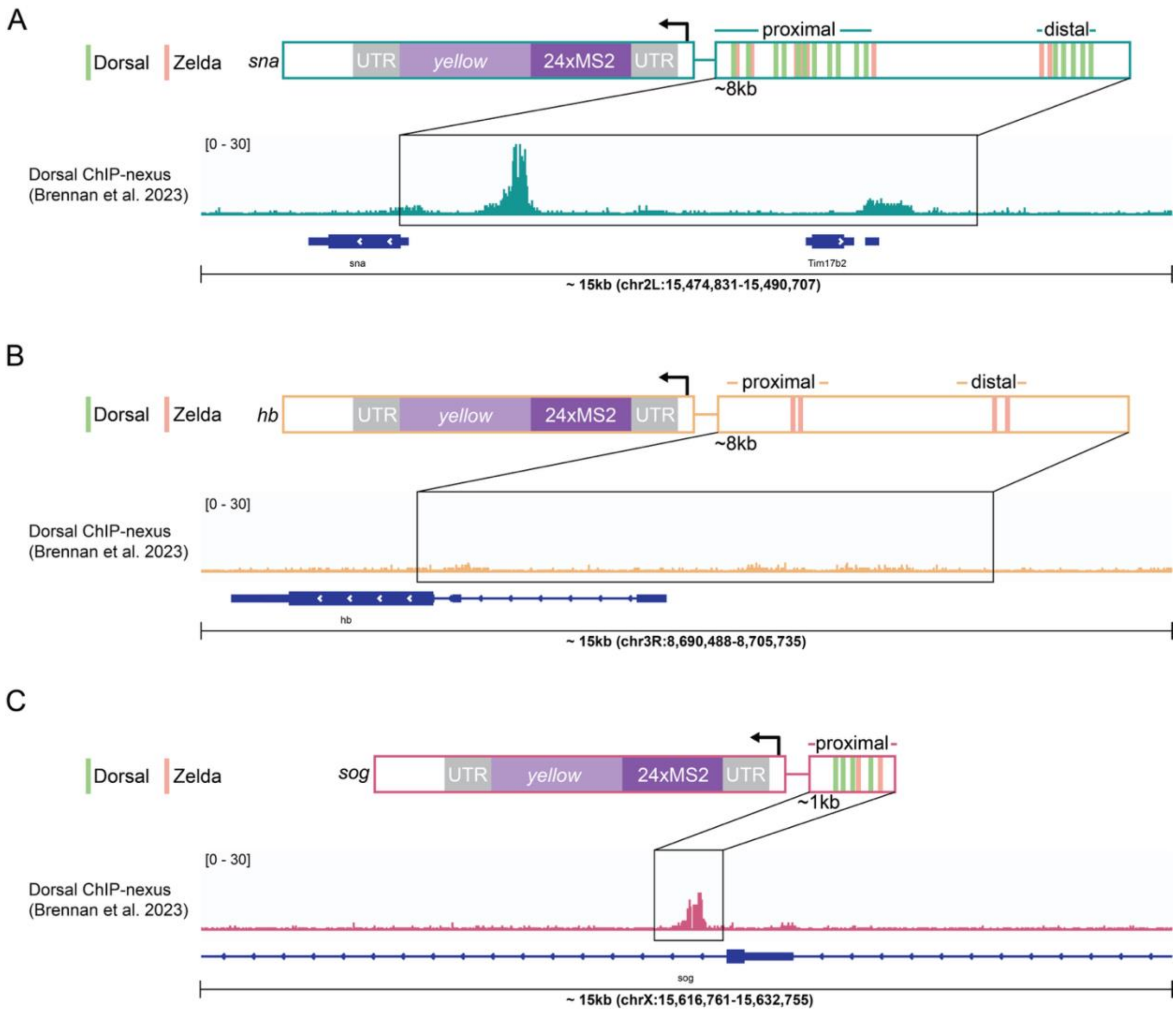

Fig. S2. Design of MS2 reporter and Dorsal occupancy in enhancer regions.

(A) Design of *snail-MS2 (BAC)* from (*42*) along with Dorsal occupancy (Dorsal ChIP-nexus from (*80*)) at the endogenous enhancer region corresponding to the region in the reporter. **(B)** Design of *hunchback-MS2 (BAC)* along with Dorsal occupancy at the endogenous enhancer region corresponding to the region in the reporter. **(C)** Design of *sog-MS2* from (*43*) along with Dorsal occupancy at the endogenous enhancer region corresponding to the region in the reporter.

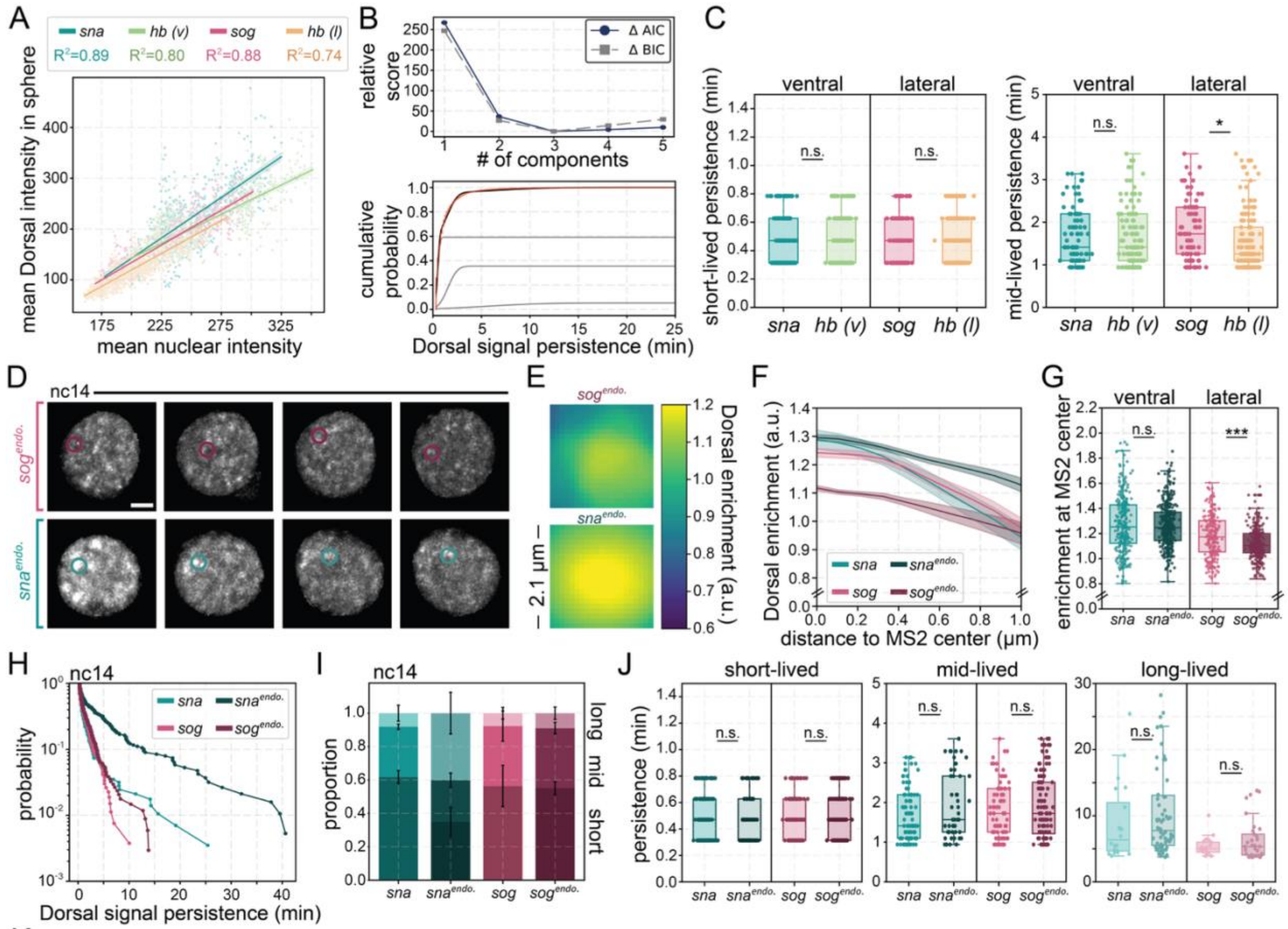

K

	short-lived	mid-lived	long-lived
sna	0.62±0.04	0.30±0.04	0.08±0.05
sna^endo.	0.35±0.09	0.25±0.04	0.40±0.12
sog	0.56±0.12	0.36±0.09	0.08±0.03
sog^endo.	0.55±0.04	0.36±0.03	0.09±0.04

Fig. S3. Dorsal hubs at transgenic and endogenous enhancers correlate with nuclear intensity and exhibit a range of persistence.

(A) Scatterplots show correlation between mean Dorsal intensity at each gene locus and mean nuclear intensity. R^2 is Pearson's Correlation. **(B)** The cumulative probability of Dorsal persistence at a locus is fit to a three-component gaussian mixture model based on minimization of Akaike Information Criterion (AIC) and Bayesian Information Criterion (BIC) (top). The fits for each component and all three together are shown (bottom). **(C)** Box plots showing the average duration of consistent hub interactions within the short- and mid-lived populations determined by a three-component gaussian-mixture model. Each persistence event is plotted. A Mann-Whitney U-test was performed to determine significance and following p-values were used: *p<0.05, **p<0.01, and ***p<0.001. **(D)** Representative maximum intensity projections of single nuclei with Dorsal-mNeonGreen in nc14. The MS2/MCP spot for each endogenous knock-in is circled in each image. The cytoplasmic Dorsal signal is masked, and contrast was adjusted independently in each row to aid visualization. Scale bars are 2 microns. **(E)** Images show average Dorsal enrichment centered at the MS2/MCP site. Images are 2.1 μm x 2.1 μm.

The color bar on the bottom shows the level of Dorsal enrichment in the image (Dorsal intensity over nuclear mean intensity). **(F)** Average radial profile with standard error centered at the MS2/MCP spot shows Dorsal enrichment as a function of distance from MS2/MCP spot center. Both transgenic enhancers (Fig. 2) and endogenous knock-ins of MS2 loops for each gene are analyzed together (see Table S1 for genotypes of embryo imaged). **(G)** Boxplots showing the distribution of the Dorsal enrichment at the center of MS2/MCP site (radius = 0.0 μm). A Mann-Whitney U-test was performed to determine significance and following p-values were used: *p<0.05, **p<0.01, and ***p<0.001. **(H)** Survival plots showing the length of time any hub is consistently present within the interaction sphere for each reporter for nc14. **(I)** Using a Gaussian Mixture Model, each hub persistence event was categorized into short-, mid-, or long-lived bins. The proportion in each bin is shown with error bars designating difference among embryo replicates. **(J)** Box plots showing the average duration of consistent hub interactions within the short-, mid-, and long-lived populations determined by a three-component gaussian-mixture model. The short-lived population is bound by distinct frames acquired 9.56 seconds apart. Each persistence event from the following nuclei are plotted. N = $sna^{endo.}$: 24 nuclei in 3 embryos and $sog^{endo.}$: 24 nuclei in 3 embryos. A Mann-Whitney U-test was performed to determine significance. **(K)** The proportion of short-, mid-, and long-lived hub persistence events are listed with their mean and standard deviation in the table.

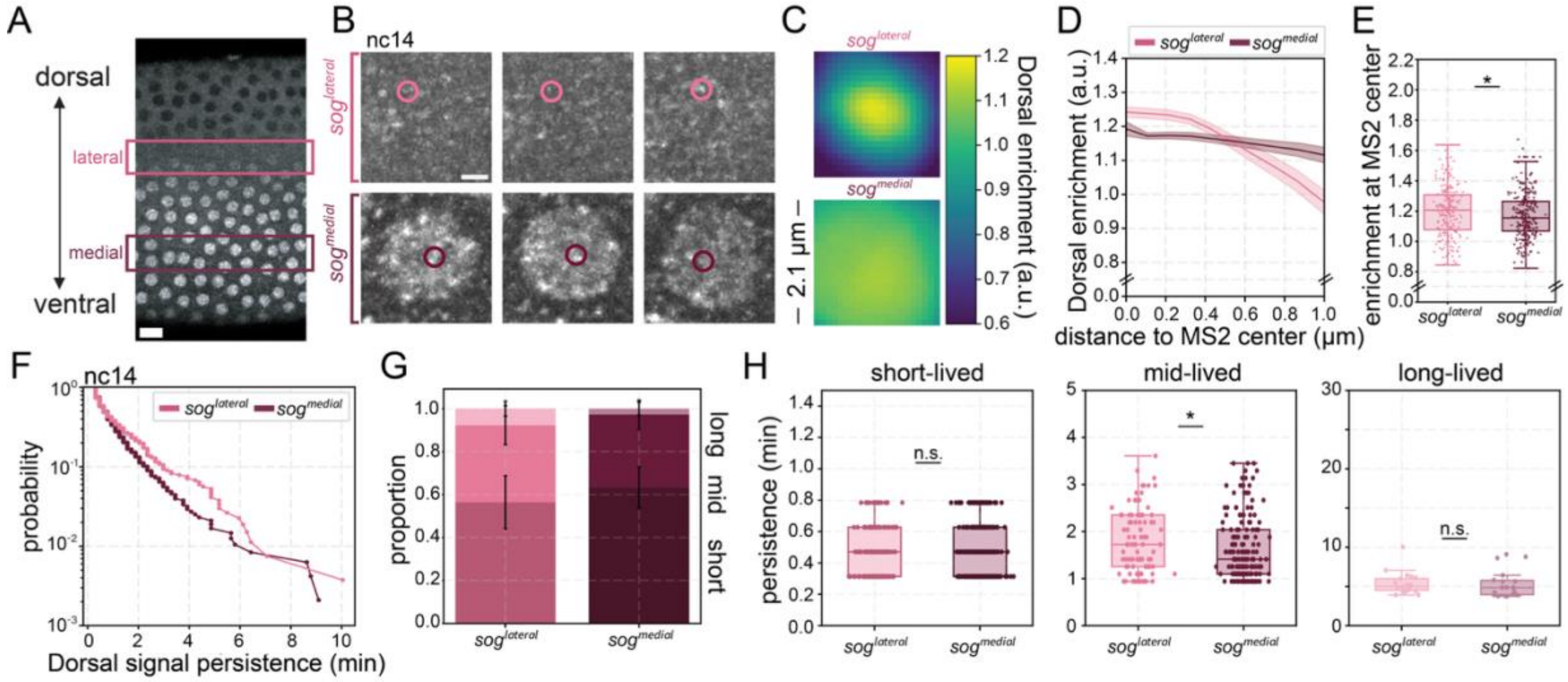

I

	short-lived	mid-lived	long-lived
$sog^{lateral}$	0.56±0.12	0.36±0.09	0.08±0.03
sog^{medial}	0.63±0.10	0.34±0.07	0.03±0.03

Fig. S4. Differences in apparent Dorsal hub properties at *sog* at different Dorsal concentrations is due to nuclear background concentration.

(A) Image of Dorsal-mNeonGreen where the 'lateral' and 'medial' *sog* nuclei were imaged (see Table S1 for genotypes of embryo imaged). **(B)** Representative maximum intensity projections of single nuclei with Dorsal-mNeonGreen in nc14. The *sog*-MS2 reporter location is circled in each image. The scale bar is 2 microns. **(C)** Images show average Dorsal enrichment centered at the MS2/MCP site. Images are 2.1 µm x 2.1 µm. The color bar on the bottom shows the level of Dorsal enrichment in the image (Dorsal intensity over nuclear mean intensity). **(D)** Average radial profile with standard error centered at the MS2/MCP spot shows Dorsal enrichment as a function of distance from MS2/MCP spot center. **(E)** Boxplots showing the distribution of the Dorsal enrichment at the center of MS2/MCP site (radius = 0.0 µm). A Mann-Whitney U-test was performed to determine significance and following p-values were used: *$p<0.05$, **$p<0.01$, and ***$p<0.001$. **(F)** Survival plots showing the length of time any hub is consistently present within the interaction sphere for each reporter for nc14. **(G)** Using a Gaussian Mixture Model, each hub persistence event was categorized into short-, mid-, or long-lived bins. The proportion in each bin is shown with error bars designating standard deviation among embryo replicates. **(H)** Box plots showing the average duration of consistent hub interactions within the short-, mid-, and long-lived populations determined by a three-component gaussian-mixture model. The short-lived population is bound by distinct frames acquired 9.56 seconds apart. Each persistence event from the following nuclei are plotted. N = $sog^{lateral}$: 18 nuclei in 3 embryos and sog^{medial}: 24 nuclei in 3 embryos. A Mann-Whitney U-test was performed to determine significance and following p-values were used: *$p<0.05$, **$p<0.01$, and ***$p<0.001$. **(I)** The proportion of short-, mid-, and long-lived hub persistence events are listed with their mean and standard deviation in the table.

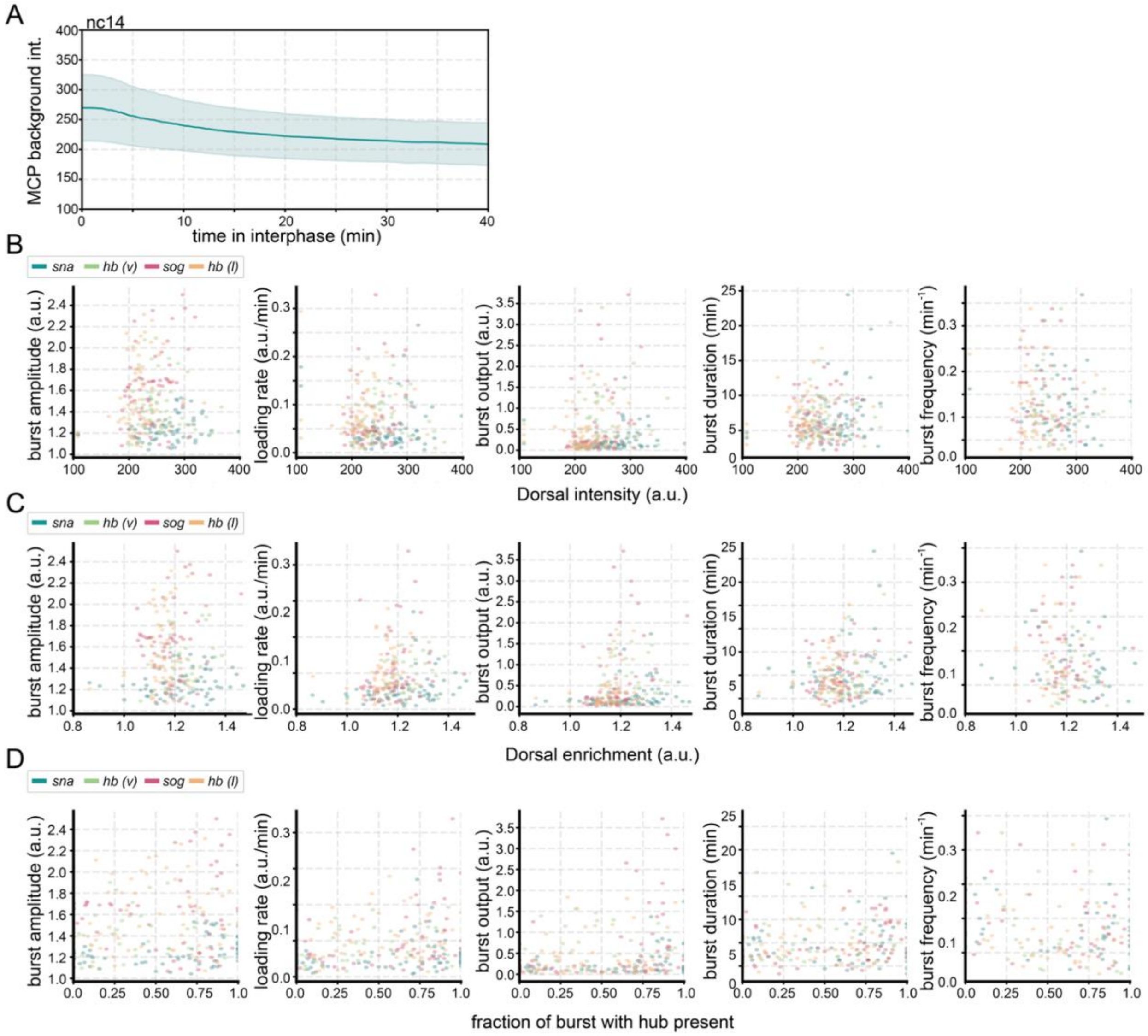

Fig. S5. Hub properties and transcription burst kinetics show minimal correlations.

(A) Average MCP background intensity within nuclei. MCP intensity decreases ~77% during nc14 due to photobleaching. **(B-D)** Scatter plots showing burst metrics (amplitude, loading rate, output, duration, frequency) with the mean Dorsal intensity **(B)**, Dorsal enrichment **(C)**, or fraction of burst with a hub present **(D)** before the burst (left), the mean Dorsal enrichment before the burst (center), or the fraction of time during a burst when a hub is persisting near the MS2/MCP site (right). Marker colors correspond to each reporter analyzed. These data points were used in the heat maps reflecting Spearman's rank correlation in Figure 3D-F.

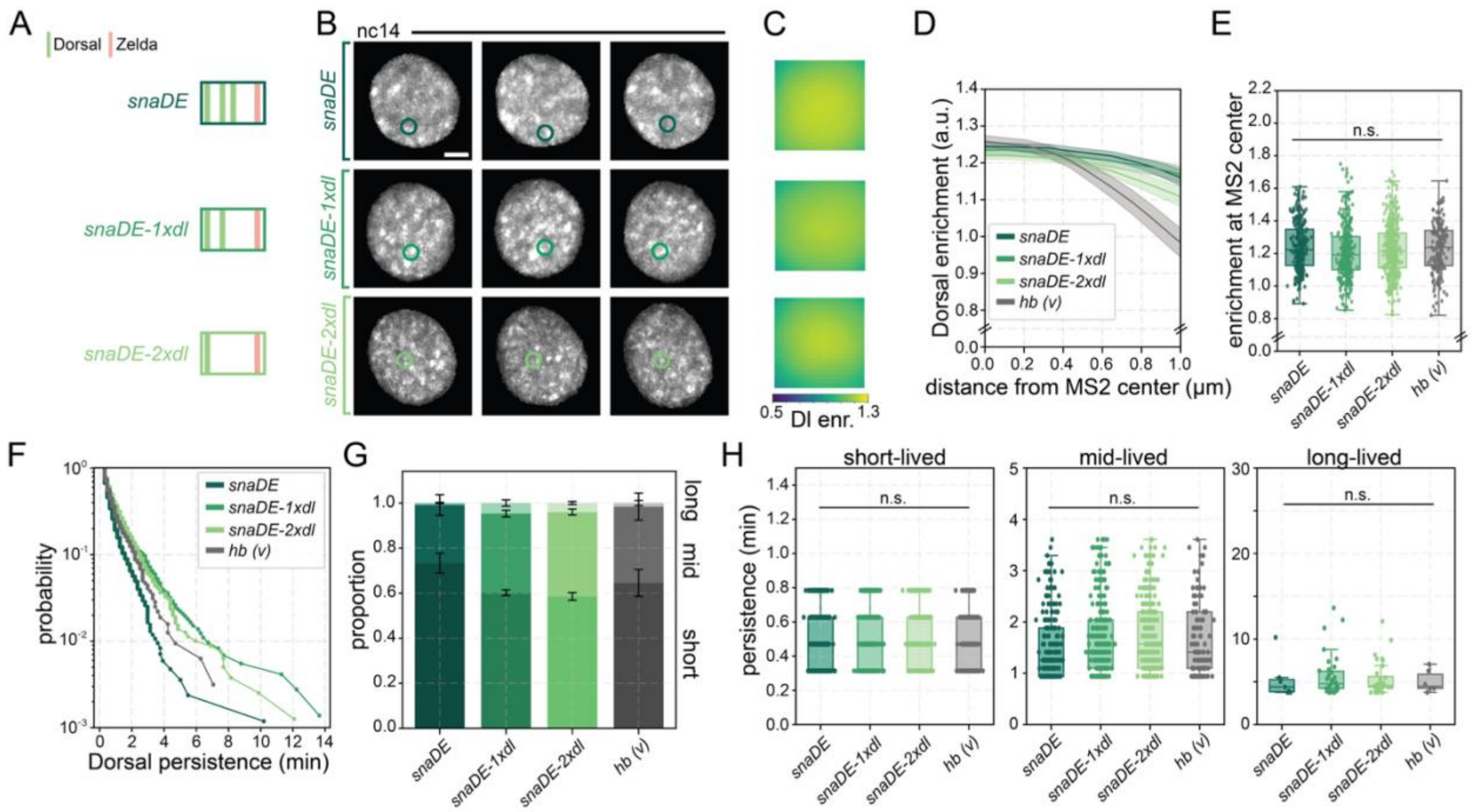

Fig. S6. Hub enrichment and signal persistence detection limits.

(A) Schematic depicting enhancers with Dorsal and Zelda binding sites for each *snail* reporter with the minimal distal enhancer of *snail* (*snaDE*), minus one Dorsal binding site (*snaDE-1xdl*), or minus two Dorsal binding site (*snaDE-2xdl*). Each enhancer was positioned just upstream of its promoter, 10 MS2 stem loops, and the *yellow* coding sequence (see Table S1 for exact genotypes of embryo imaged). Each of these *snail* reporters were imaged along the ventral surface in nc14. **(B)** Representative maximum intensity projections of single nuclei with Dorsal-mNeonGreen in nc14. The MS2/MCP site is circled in each image. The cytoplasmic Dorsal signal is masked, and contrast was adjusted independently in each row to aid visualization. Scale bars are 2 microns. **(C)** Images show average Dorsal enrichment centered at the MS2/MCP site. Images are 2.1 µm x 2.1 µm. The color bar on the bottom shows the level of Dorsal enrichment in the image (Dorsal intensity over nuclear mean intensity). **(D)** Average radial profile with standard error centered at the MS2/MCP spot shows Dorsal enrichment as a function of distance from MS2/MCP spot center. **(E)** Boxplots showing the distribution of the Dorsal enrichment at the center of MS2/MCP site (radius = 0.0 µm). A Mann-Whitney U-test was performed to determine significance. **(F)** Survival plots showing the length of time any hub is consistently present within the interaction sphere for each reporter for nc14. **(G)** After applying a Gaussian Mixture Model, each hub persistence event was categorized into short-, mid-, or long-lived bins. The proportion in each bin is shown with error bars designating standard deviation among embryo replicates. **(H)** Box plots showing the average duration of consistent hub interactions within the short-, mid-, and long-lived populations determined by a three-component gaussian-mixture model. The short-lived population is bound by distinct frames acquired 9.56 seconds apart. Each persistence event from the following nuclei are plotted. N = *snaDE:* 28 nuclei in 3 embryos; *snaDE-1xdl:* 33 nuclei in 3 embryos; *snaDE-2xdl:* 44 nuclei in 4 embryos; and *hb (v):* 18 nuclei in 4 embryos. A Mann-Whitney U-test was performed to determine significance and following p-values were used: *$p<0.05$, **$p<0.01$, and ***$p<0.001$.

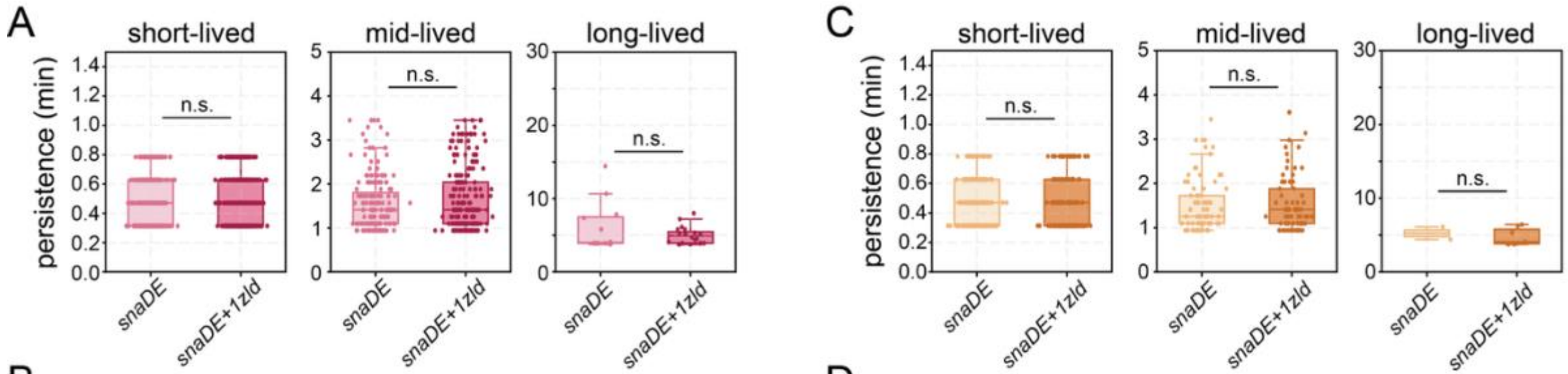

B

Dorsal	short-lived	mid-lived	long-lived
snaDE	0.66±0.04	0.32±0.04	0.02±0.01
snaDE+1zld	0.64±0.10	0.33±0.08	0.03±0.03

D

Zelda	short-lived	mid-lived	long-lived
snaDE	0.72±0.10	0.28±0.10	0.01±0.01
snaDE+1zld	0.73±0.03	0.25±0.04	0.02±0.01

Fig. S7. Dorsal and Zelda persistence are moderately impacted from a single Zelda binding site addition.

(A, C) Box plots showing the average duration of consistent Dorsal **(A)** or Zelda **(C)** hub interactions within the short-, mid-, and long-lived populations determined by a three-component gaussian-mixture model. The short-lived population is bound by distinct frames acquired 9.56 seconds apart. Each persistence event from all nuclei are plotted. A Mann-Whitney U-test was performed to determine significance and following p-values were used: *p<0.05, **p<0.01, and ***p<0.001. **(B, D)** The proportion of short-, mid-, and long-lived Dorsal **(B)** or Zelda **(D)** hub persistence events are listed with their means and standard deviations in the table.

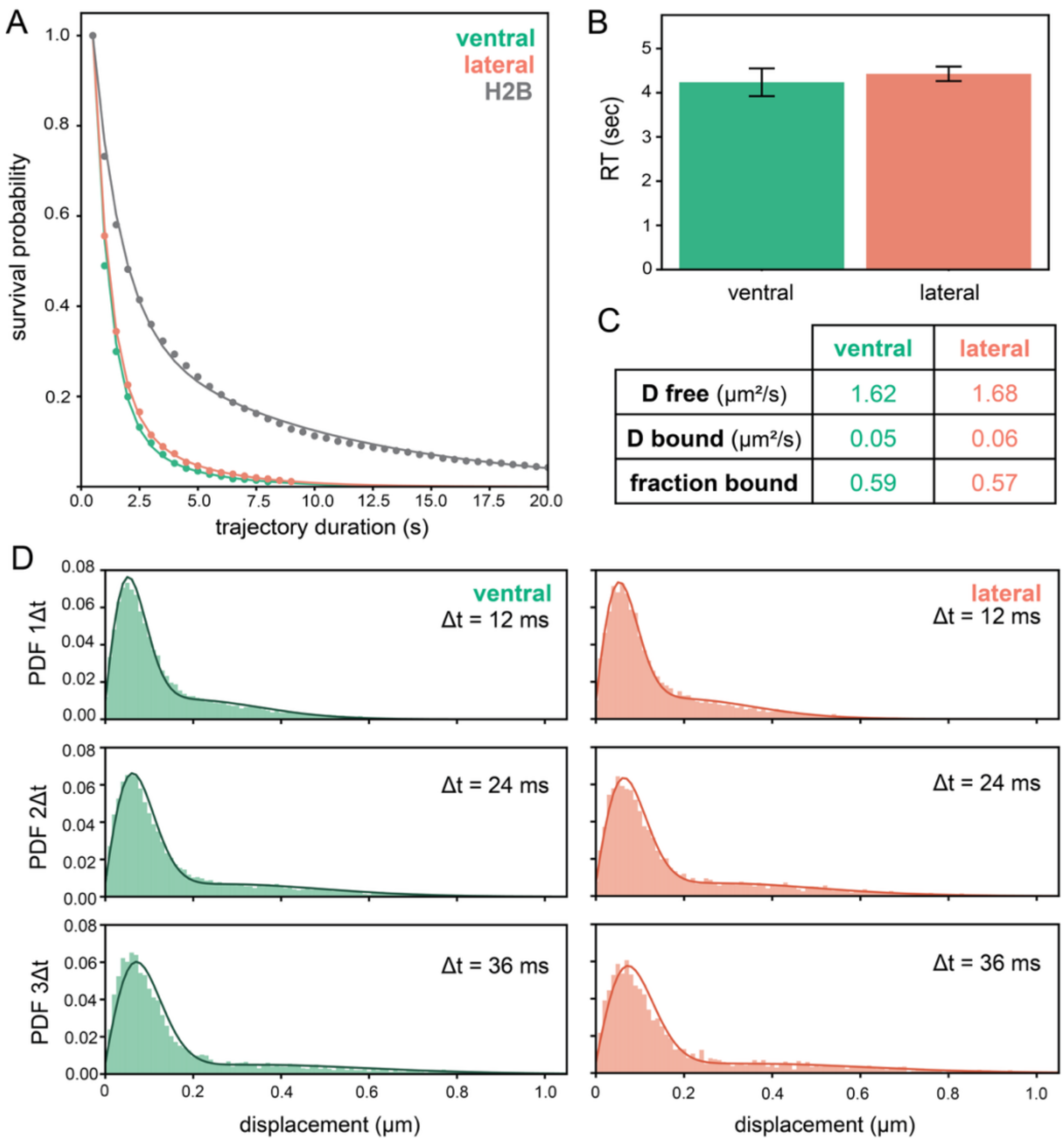

Fig. S8. Single molecule tracking (SMT) of Dorsal shows minimal differences on the ventral and lateral regions.

(A) Survival probabilities of single-molecule trajectories at 500 msec exposure times for at least three embryos for Dorsal-mEos4a ventrally positioned (N = 978 trajectories, 5 unique embryos), Dorsal-mEos4a laterally positioned (N = 1214 unique trajectories, 4 unique embryos), and H2B-mEos4a (N = 3098 trajectories, 3 unique embryos)**. (B)** Doral residence times after photobleaching correction for ventral (4.24 ± 0.32 sec) and lateral (4.43 ± 0.17 sec) positions. **(C)** Spot-On diffusion coefficient estimates for free and bound populations. **(D)** Probability density functions (PDFs) of the trajectory displacements of ventral (N = 10968 trajectories, 4 unique embryos) and lateral (N = 5824 trajectories, 11 unique embryos) Dorsal-mEos4a across three time intervals.

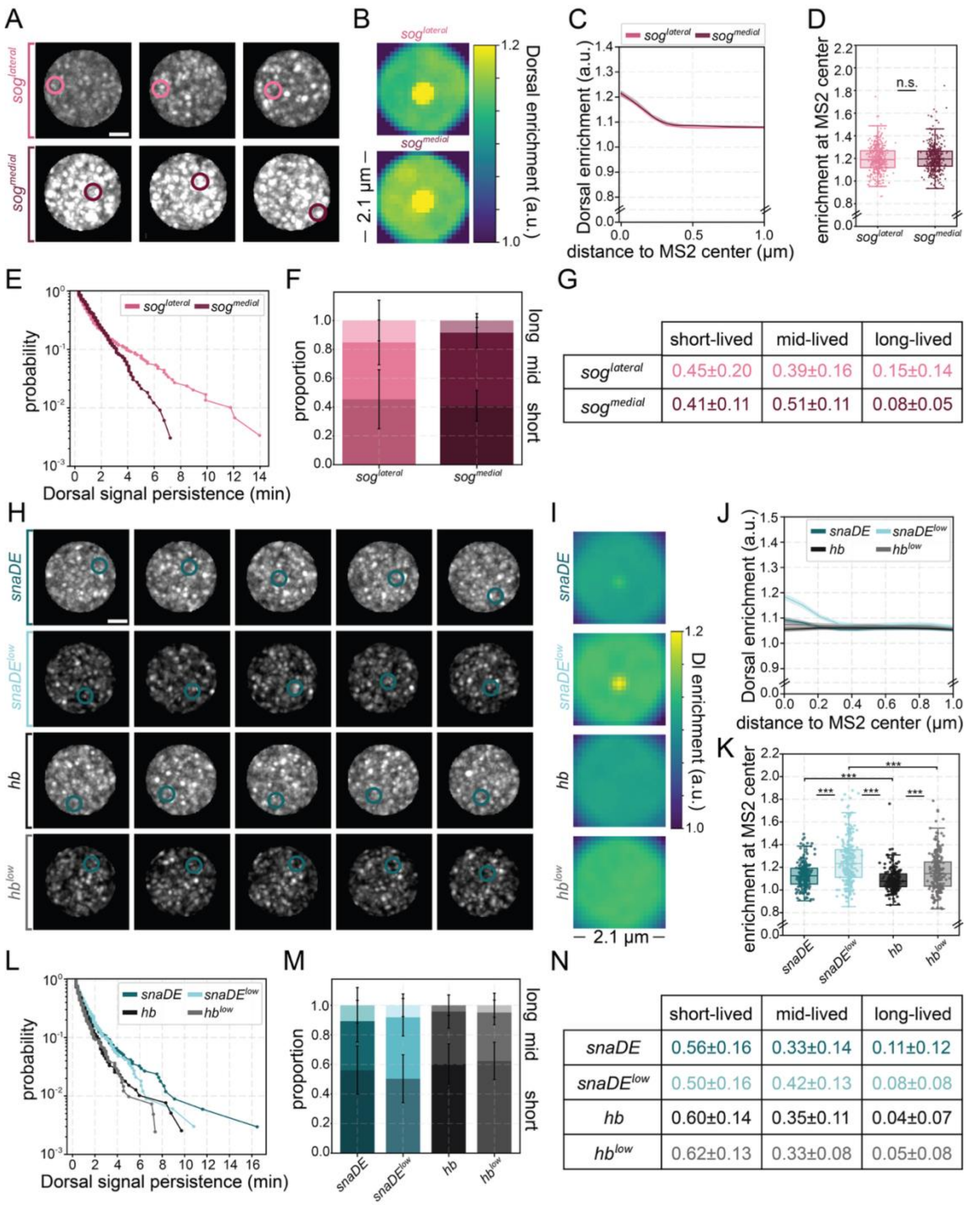

	short-lived	mid-lived	long-lived
sog^lateral	0.45±0.20	0.39±0.16	0.15±0.14
sog^medial	0.41±0.11	0.51±0.11	0.08±0.05

	short-lived	mid-lived	long-lived
snaDE	0.56±0.16	0.33±0.14	0.11±0.12
snaDE^low	0.50±0.16	0.42±0.13	0.08±0.08
hb	0.60±0.14	0.35±0.11	0.04±0.07
hb^low	0.62±0.13	0.33±0.08	0.05±0.08

Fig. S9. Computational modeling shows the effect of nuclear concentration and imaging conditions on hub properties.

(A) Representative maximum intensity projections of simulated single nuclei with Dorsal signal in grey. The binding site cluster is circled in each image. Contrast is scaled equally for all conditions. Scale bars are 2 microns. **(B)** Images show simulated average Dorsal enrichment centered at the binding site clusters. Images are 2.1 µm x 2.1 µm. The color bar on the bottom shows the level of Dorsal enrichment in the image (Dorsal intensity over nuclear mean intensity).

(C) Average radial profile with standard error centered at the binding site cluster shows Dorsal enrichment as a function of distance from spot center. **(D)** Boxplots showing the distribution of the Dorsal enrichment at the center of the binding site cluster (radius = 0.0 µm). A Mann-Whitney U-test was performed to determine significance and following p-values were used: *p<0.05, **p<0.01, and ***p<0.001. **(E)** Survival plots showing the length of time any hub is consistently present within the interaction sphere for each reporter. **(F)** The proportion of long-, mid-, and short-lived persistent hub formation in each bin is shown with error bars designating standard deviation among embryo replicates. **(G)** The proportion of short-, mid-, and long-lived hub persistence events are listed with their mean and standard deviation in the table. **(H)** Representative maximum intensity projections of simulated single nuclei with Dorsal signal in grey. The binding site cluster is circled in each image. Contrast is scaled equally for all conditions. Scale bars are 2 microns. Simulations show *snaDE* or *hb* visualized with regular imaging conditions or with lowered background and noise. **(I)** Images show simulated average Dorsal enrichment centered at the binding site clusters. Images are 2.1 µm x 2.1 µm. The color bar on the bottom shows the level of Dorsal enrichment in the image (Dorsal intensity over nuclear mean intensity). **(J)** Average radial profile with standard error centered at the binding site cluster shows Dorsal enrichment as a function of distance from spot center. **(K)** Boxplots showing the distribution of the Dorsal enrichment at the center of the binding site cluster (radius = 0.0 µm). A Mann-Whitney U-test was performed to determine significance and following p-values were used: *p<0.05, **p<0.01, and ***p<0.001. **(L)** Survival plots showing the length of time any hub is consistently present within the interaction sphere for each reporter. **(M)** The proportion of long-, mid-, and short-lived persistent hub formation in each bin is shown with error bars designating standard deviation among embryo replicates. **(N)** The proportion of short-, mid-, and long-lived hub persistence events are listed with their mean and standard deviation in the table.

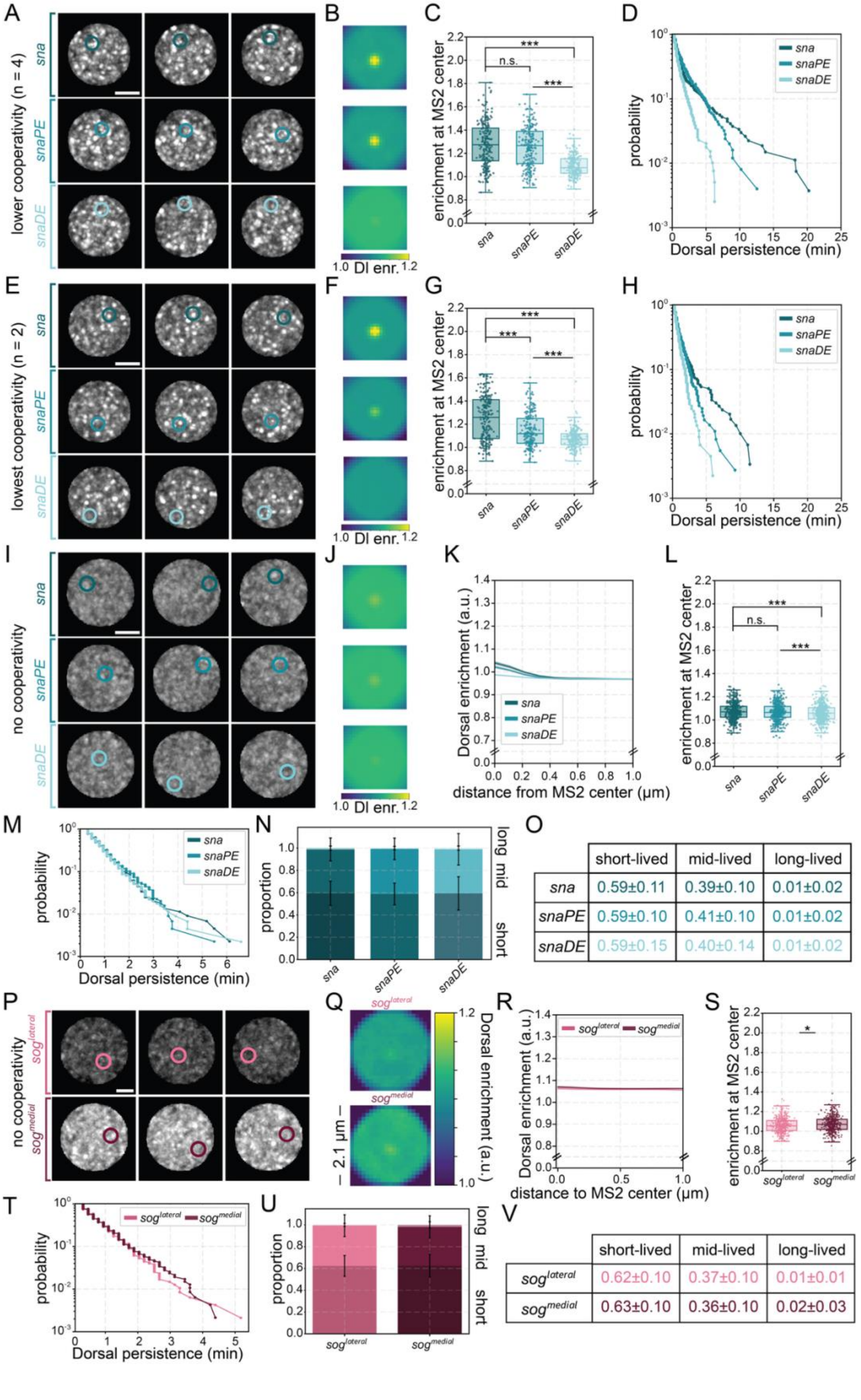

	short-lived	mid-lived	long-lived
sna	0.59±0.11	0.39±0.10	0.01±0.02
snaPE	0.59±0.10	0.41±0.10	0.01±0.02
snaDE	0.59±0.15	0.40±0.14	0.01±0.02

	short-lived	mid-lived	long-lived
$sog^{lateral}$	0.62±0.10	0.37±0.10	0.01±0.01
sog^{medial}	0.63±0.10	0.36±0.10	0.02±0.03

Fig. S10. Computational modeling without cooperativity fails to recapitulate Dorsal hub properties.

(A, E, I P) Representative maximum intensity projections of simulated single nuclei with cooperative binding (Hill-like = 2 **(A),** 4 **(E)**) or without cooperative binding included in computation **(I, P)** (Dorsal signal in grey). The binding site cluster is circled in each image. Contrast is scaled equally for all conditions. Scale bars are 2 microns. **(B, F, J, Q)** Images show simulated average Dorsal enrichment centered at the binding site clusters. Images are 2.1 μm x 2.1 μm. The color bar on the bottom shows the level of Dorsal enrichment in the image (Dorsal intensity over nuclear mean intensity). **(C, G, L, S)** Boxplots showing the distribution of the Dorsal enrichment at the center of the binding site cluster (radius = 0.0 μm). A Mann-Whitney U-test was performed to determine significance and following p-values were used: *p<0.05, **p<0.01, and ***p<0.001. **(D, H, M, T)** Survival plots showing the length of time any hub is consistently present within the interaction sphere for each reporter. **(K, R)** Average radial profile with standard error centered at the binding site cluster shows Dorsal enrichment as a function of distance from spot center. **(N, U)** The proportion of long-, mid-, and short-lived persistent hub formation in each bin is shown with error bars designating standard deviation among embryo replicates. **(O, V)** The proportion of short-, mid-, and long-lived hub persistence events are listed with their mean and standard deviation in the table.

Table S1. Details of *Drosophila* lines used.

**Shorthand**	**Genotype***	**Source**	**Appears in Figures**
Dorsal-mNeonGreen	yw; Dorsal-mNeonGreen/CyO;	(*41*)	1, 2, 3, 4, S1, S3, S4, S5, S6, S7, S8
Dorsal-mEos4a	yw; Dorsal-mEos4a/CyO;	(*41*)	6, S8
mNeonGreen-Zelda	mNeonGreen-Zelda;;	(*52*)	5, S7
MCP-mCherry	nos>MCP-mCherry (III)	(*54*)	2, 3, 4, S3, S4, S5, S6, S7, S8
MCP-mCherry	enosx2>MCP-mCherry (II)	(*41*)	5, S7
sna	sna-24xMS2 (BAC)	(*42*)	2, 3, S3, S5
hb	hb-24xMS2 (BAC)	(*42*)	2, 3, 4, S3, S5
sog, *sog*^{lateral}, *sog*^{medial}	sog-24xMS2 (BAC)	(*43*)	2, 3, S3, S4, S5
sna$^{endo.}$	sna-24xMS2 (endogenous 3’)	(*44*)	S3
sog$^{endo.}$	sog-24xMS2 (endogenous 5’)	Gift from Bomyi Lim	S3
snaPE	snaPE > snaPr > 24xMS2-yellow	Gift from Bomyi Lim	4
snaDE	snaDE > snaPr > 24xMS2 - yellow	(*39*)	4, 5, S7
snaDE	snaDE > snaPr > 10xMS2 - yellow	(*49*)	S6
snaDE - 1xdl	snaDE del dl1 > snaPr > 10xMS2 - yellow	(*49*)	S6
snaDE - 2xdl	snaDE del dl1/2 > snaPr > 10xMS2 - yellow	(*49*)	S6
snaDE + 1 zld	snaDE + 1 Zld 5’ > snaPr > 24xMS2 - yellow	(*39*)	5, S7

* All non-endogenous MS2 reporters inserted into VK33 on Chromosome III

Table S2. Simulation parameters.

**Category**	**Parameter**	**Value**
General Parameters	Dorsal concentration from (*54*)	Ventral = 500 nM
		Medial = 406 nM
		Lateral = 219 nM
	Nucleus diameter	5 µm
	Molecule diameter	15 nm
	Simulation time step	10 ms
	Display time step	10 s
Binding sites	Number (post-replication) from (*50*)	7000
	Endogenous sites per cluster	2 (60%), 10 (25%), 20 (10%), 30 (4%), 40 (1%)
	Endogenous site cluster diameter	3 px = 324 nm
	Diffusion coefficient	0.05 ± 0.03 $\mu m^2/s$ (0.001 µm jiggle)
	Hurst exponent	0.25
	Synthetic sites per cluster (post-replication)	*snail* = 32 (in a 1, 3 or 5 px diameter)
		snailPE = 22 (in a 1, 2 or 4 px diameter)
		snailDE = 6 (in a 1, 2 or 3 px diameter)
		sog = 8 (in a 1, 2 or 3 px diameter)
Three-state diffusion	Initialization	50% free, 50% non-specifically bound

model	Free diffusion coefficient	1.7 ± 0.45 $\mu m^2/s$
	Switching rate (free to non-specific binding)	1 Hz
	Non-specific residence time	0.5 s
	Binding probability, k_{on}	0.005
	Capture radius	50 nm
Binding site strength distribution	Base residence time, $1/k_{off}$	5 ± 1 s
	Endogenous site residence time (multipliers of base residence time)	0.06x (50%), 0.2x (27%), 0.5x (15%), 1x (6%), 2.4x (2%)
	Synthetic site residence time (multipliers of base residence time)	*snail*/*snailPE*/*snailDE* = 0.2x
		hunchback = 0.000001x
		sog = 0.5x
	Hill-like coefficient, n	2 and 4 (lowered cooperativity), or 100 (with cooperativity)

Movie S1. Dorsal hub formation in nc13 and 14.

Movies showing Dorsal-mNeonGreen (gray) in nuclei along the lateral (left) or ventral (right) surface of a *Drosophila* embryo in nc13 and nc14. Each movie is a 3D rendering with equivalent contrast limits.

Movie S2. Dorsal hubs at the lateral transition point.

Movie showing Dorsal-mNeonGreen (gray) in 3D rendered nuclei along the lateral transition point of a *Drosophila* embryo in nc13 and nc14.

Movie S3. Dorsal hub segmentation example.

Example of hub segmentation (colored labels) within ventral nuclei of Dorsal-mNeonGreen (gray). Movie starts on a single z-slice then moves through the whole nucleus in z.

Movie S4. Dorsal hubs at target and non-target genes in nc13.

Example 3D rendering of nuclei through time showing Dorsal-mNeonGreen (gray) at MS2 spots (circled) for *sna, hb (v), sog,* and *hb (l)* in nc13. For visualization purposes, cytoplasm has been removed and set to zero, and contrast limits are individually adjusted for ventral or lateral nuclei.

Movie S5. Dorsal hubs at target and non-target genes in nc14.

Example 3D rendering of nuclei through time showing Dorsal-mNeonGreen (gray) at MS2 spots (circled) for *sna, hb (v), sog,* and *hb (l)* in nc14. For visualization purposes, cytoplasm has been removed and set to zero, and contrast limits are individually adjusted for ventral or lateral nuclei.

Movie S6. Example of MS2/MCP tracking at *sog*.

Example of 3D MS2 tracking in time within a single nucleus. Gray channel shows MCP-mCherry distribution in the nucleus. For visualization purposes, cytoplasm has been removed and set to zero. A pink circle marks the spot of nascent transcription of *sog*-MS2. The left shows a top-down (X-Y) view while the right shows a side (Y-Z) view.

Movie S7. Dorsal hubs at endogenous target genes.

Example 3D rendering of nuclei through time showing Dorsal-mNeonGreen (gray) at MS2 spots (circled) for *snail (endogenous)* and *sog (endogenous)* in nc14. For visualization purposes, cytoplasm has been removed and set to zero, and contrast limits are individually adjusted for ventral or lateral nuclei.

Movie S8. Dorsal hubs at *sog* at different concentrations.

Example 3D rendering of nuclei through time showing Dorsal-mNeonGreen (gray) at MS2 spots (circled) for *sog (lateral)* and *sog (medial)* in nc14. Contrast limits are equivalent across conditions.

Movie S9. Dorsal hubs at various *snail* enhancers.

Example 3D rendering of nuclei through time showing Dorsal-mNeonGreen (gray) at MS2 spots (circled) for *sna, snaPE,* and *snaDE* in nc14. For visualization purposes, cytoplasm has been removed and set to zero. Contrast limits are equivalent across conditions.

Movie S10. Dorsal hubs when binding sites are removed.

Example 3D rendering of nuclei through time showing Dorsal-mNeonGreen (gray) at MS2 spots (circled) for *snaDE, snaDE-1xdl,* and *snaDE-2xdl* in nc14. For visualization purposes, cytoplasm has been removed and set to zero. Contrast limits are equivalent across conditions.

Movie S11. Dorsal hubs upon the addition of a Zelda binding site.

Example 3D rendering of nuclei through time showing Dorsal-mNeonGreen (gray) at MS2 spots (circled) for *snaDE* and *snaDE+1zld* in nc14. For visualization purposes, cytoplasm has been removed and set to zero. Contrast limits are equivalent across conditions.

Movie S12. Zelda hubs upon the addition of a Zelda binding site.

Example 3D rendering of nuclei through time showing mNeonGreen-Zelda (gray) at MS2 spots (circled) for *snaDE* and *snaDE+1zld* in nc14. For visualization purposes, cytoplasm has been removed and set to zero. Contrast limits are equivalent across conditions.

REFERENCES

1. A. P. Boka, A. Mukherjee, M. Mir, Single-molecule tracking technologies for quantifying the dynamics of gene regulation in cells, tissue and embryos. *Development* **148**, dev199744 (2021).

2. F. Lu, T. Lionnet, Transcription factor dynamics. *Cold Spring Harb. Perspect. Biol.* **13**, a040949 (2021).

3. G. Hayward-Lara, M. D. Fischer, M. Mir, Dynamic microenvironments shape nuclear organization and gene expression. *Curr. Opin. Genet. Dev.* **86**, 102177 (2024).

4. K. Wagh, D. A. Stavreva, A. Upadhyaya, G. L. Hager, Transcription factor dynamics: One molecule at a time. *Annu. Rev. Cell Dev. Biol.* **39**, 277–305 (2023).

5. A. Tsai, A. K. Muthusamy, M. R. P. Alves, L. D. Lavis, R. H. Singer, D. L. Stern, J. Crocker, Nuclear microenvironments modulate transcription from low-affinity enhancers. *eLife* **6**, e28975 (2017).

6. A. S. Hansen, A. Amitai, C. Cattoglio, R. Tjian, X. Darzacq, Guided nuclear exploration increases CTCF target search efficiency. *Nat. Chem. Biol.* **16**, 257–266 (2020).

7. A. Navalkar, M. Eppert, B. R. Sabari, T. Mittag, Density transitions in the regulation of transcription. *Mol. Cell* **86**, 567–584 (2026).

8. M. Mir, A. Reimer, J. E. Haines, X.-Y. Li, M. Stadler, H. Garcia, M. B. Eisen, X. Darzacq, Dense Bicoid hubs accentuate binding along the morphogen gradient. *Genes Dev.* **31**, 1784–1794 (2017).

9. M. Mir, M. R. Stadler, S. A. Ortiz, C. E. Hannon, M. M. Harrison, X. Darzacq, M. B. Eisen, Dynamic multifactor hubs interact transiently with sites of active transcription in Drosophila embryos. *eLife* **7**, e40497 (2018).

10. B. R. Sabari, A. Dall'Agnese, A. Boija, I. A. Klein, E. L. Coffey, K. Shrinivas, B. J. Abraham, N. M. Hannett, A. V. Zamudio, J. C. Manteiga, C. H. Li, Y. E. Guo, D. S. Day, J. Schuijers, E. Vasile, S. Malik, D. Hnisz, T. I. Lee, I. I. Cisse, R. G. Roeder, P. A. Sharp, A. K. Chakraborty,

R. A. Young, Coactivator condensation at super-enhancers links phase separation and gene control. *Science* **361**, eaar3958 (2018).

11. K. Kawasaki, T. Fukaya, Functional coordination between transcription factor clustering and gene activity. *Mol. Cell* **83**, 1605–1622.e9 (2023).

12. W.-K. Cho, J.-H. Spille, M. Hecht, C. Lee, C. Li, V. Grube, I. I. Cisse, Mediator and RNA polymerase II clusters associate in transcription-dependent condensates. *Science* **361**, 412–415 (2018).

13. A. Boija, I. A. Klein, B. R. Sabari, A. Dall'Agnese, E. L. Coffey, A. V. Zamudio, C. H. Li, K. Shrinivas, J. C. Manteiga, N. M. Hannett, B. J. Abraham, L. K. Afeyan, Y. E. Guo, J. K. Rimel, C. B. Fant, J. Schuijers, T. I. Lee, D. J. Taatjes, R. A. Young, Transcription factors activate genes through the phase-separation capacity of their activation domains. *Cell* **175**, 1842–1855.e16 (2018).

14. I. I. Cisse, I. Izeddin, S. Z. Causse, L. Boudarene, A. Senecal, L. Muresan, C. Dugast-Darzacq, B. Hajj, M. Dahan, X. Darzacq, Real-time dynamics of RNA polymerase II clustering in live human cells. *Science* **341**, 664–667 (2013).

15. M.-T. Wei, Y.-C. Chang, S. F. Shimobayashi, Y. Shin, A. R. Strom, C. P. Brangwynne, Nucleated transcriptional condensates amplify gene expression. *Nat. Cell Biol.* **22**, 1187–1196 (2020).

16. M. Du, S. H. Stitzinger, J.-H. Spille, W.-K. Cho, C. Lee, M. Hijaz, A. Quintana, I. I. Cissé, Direct observation of a condensate effect on super-enhancer controlled gene bursting. *Cell* **187**, 2595–2598 (2024).

17. A. Tsai, M. R. Alves, J. Crocker, Multi-enhancer transcriptional hubs confer phenotypic robustness. *eLife* **8**, e45325 (2019).

18. S. Chong, C. Dugast-Darzacq, Z. Liu, P. Dong, G. M. Dailey, C. Cattoglio, A. Heckert, S. Banala, L. Lavis, X. Darzacq, R. Tjian, Imaging dynamic and selective low-complexity domain interactions that control gene transcription. *Science* **361**, eaar2555 (2018).

19. M. Mazzocca, A. Loffreda, E. Colombo, T. Fillot, D. Gnani, P. Falletta, E. Monteleone, S. Capozi, E. Bertrand, G. Legube, Z. Lavagnino, C. Tacchetti, D. Mazza, Chromatin organization drives the search mechanism of nuclear factors. *Nat. Commun.* **14**, 6433 (2023).

20. A. Mukherjee, M. Kapoor, K. Shankta, S. Fallacaro, R. D. Carter, G. Hayward-Lara, P. Ratchasanmuang, Y. I. Haloush, M. Mir, A single cluster of RNA polymerase II molecules is stably associated with active genes. bioRxiv 637507 [Preprint] (2025). https://doi.org/10.1101/2025.02.10.637507.

21. S. Fallacaro, A. Mukherjee, P. Ratchasanmuang, J. Zinski, Y. I. Haloush, K. Shankta, M. Mir, A fine kinetic balance of interactions directs transcription factor hubs to genes. bioRxiv 589811 [Preprint] (2024). https://doi.org/10.1101/2024.04.16.589811.

22. C. Santa-Cruz Mateos, C. Roussos, F. J. de Haro Arbona, J. Falo-Sanjuan, S. Bray, Direct observation of Notch signalling induced transcription hubs mediating gene-expression responses. bioRxiv 663121 [Preprint] (2025). https://doi.org/10.1101/2025.07.04.663121.

23. S. F. Banani, H. O. Lee, A. A. Hyman, M. K. Rosen, Biomolecular condensates: Organizers of cellular biochemistry. *Nat. Rev. Mol. Cell Biol.* **18**, 285–298 (2017).

24. A. Musacchio, On the role of phase separation in the biogenesis of membraneless compartments. *EMBO J.* **41**, e109952 (2022).

25. G. Pei, H. Lyons, P. Li, B. R. Sabari, Transcription regulation by biomolecular condensates. *Nat. Rev. Mol. Cell Biol.* **26**, 213–236 (2025).

26. M. M. Gaskill, I. V. Soluri, A. E. Branks, A. P. Boka, M. R. Stadler, K. Vietor, H.-Y. S. Huang, T. J. Gibson, A. Mukherjee, M. Mir, S. A. Blythe, M. M. Harrison, Localization of the *Drosophila* pioneer factor GAF to subnuclear foci is driven by DNA binding and required to silence satellite repeat expression. *Dev. Cell* **58**, 1610–1624.e8 (2023).

27. A. Patil, A. R. Strom, J. A. Paulo, C. K. Collings, K. M. Ruff, M. K. Shinn, A. Sankar, K. S. Cervantes, T. Wauer, J. D. St Laurent, G. Xu, L. A. Becker, S. P. Gygi, R. V. Pappu, C. P.

Brangwynne, C. Kadoch, A disordered region controls cBAF activity via condensation and partner recruitment. *Cell* **186**, 4936–4955.e26 (2023).

28. N. M. Chabot, R. Purkanti, A. Del Panta Ridolfi, D. D. Nogare, H. Oda, H. Kimura, F. Jug, A. Dal Co, N. L. Vastenhouw, Local DNA compaction creates TF-DNA clusters that enable transcription. bioRxiv 605169 [Preprint] (2024). https://doi.org/10.1101/2024.07.25.605169.

29. M. Ugolini, M. A. Kerlin, K. Kuznetsova, H. Oda, H. Kimura, N. L. Vastenhouw, Transcription bodies regulate gene expression by sequestering CDK9. *Nat. Cell Biol.* **26**, 604–612 (2024).

30. S. Dongre, N. L. Vastenhouw, Specificity in clustering of gene-specific transcription factors is encoded in the genome. *Nucleic Acids Res.* **53**, gkaf625 (2025).

31. T. Mittag, R. V. Pappu, A conceptual framework for understanding phase separation and addressing open questions and challenges. *Mol. Cell* **82**, 2201–2214 (2022).

32. S. Yamada, P. H. Whitney, S.-K. Huang, E. C. Eck, H. G. Garcia, C. A. Rushlow, The *Drosophila* pioneer factor Zelda modulates the nuclear microenvironment of a dorsal target enhancer to potentiate transcriptional output. *Curr. Biol.* **29**, 1387–1393.e5 (2019).

33. F. J. DeHaro-Arbona, C. Roussos, S. Baloul, J. Townson, M. J. Gómez Lamarca, S. Bray, Dynamic modes of Notch transcription hubs conferring memory and stochastic activation revealed by live imaging the co-activator Mastermind. *eLife* **12**, RP92083 (2024).

34. W. Pomp, J. V. W. Meeussen, T. L. Lenstra, Transcription factor exchange enables prolonged transcriptional bursts. *Mol. Cell* **84**, 1036–1048.e9 (2024).

35. B. T. Donovan, A. Huynh, D. A. Ball, H. P. Patel, M. G. Poirier, D. R. Larson, M. L. Ferguson, T. L. Lenstra, Live-cell imaging reveals the interplay between transcription factors, nucleosomes, and bursting. *EMBO J.* **38**, e100809 (2019).

36. C. A. Rushlow, K. Han, J. L. Manley, M. Levine, The graded distribution of the dorsal morphogen is initiated by selective nuclear transport in *Drosophila*. *Cell* **59**, 1165–1177 (1989).

37. A. E. Schloop, P. U. Bandodkar, G. T. Reeves, Formation, interpretation, and regulation of the *Drosophila* Dorsal/NF-κB gradient. *Curr. Top. Dev. Biol.* **137**, 143–191 (2020).

38. R. Munshi, J. Ling, S. Ryabichko, E. F. Wieschaus, T. Gregor, Transcription factor clusters as information transfer agents. *Sci. Adv.* **11**, eadp3251 (2025).

39. J. Dufourt, A. Trullo, J. Hunter, C. Fernandez, J. Lazaro, M. Dejean, L. Morales, S. Nait-Amer, K. N. Schulz, M. M. Harrison, C. Favard, O. Radulescu, M. Lagha, Temporal control of gene expression by the pioneer factor Zelda through transient interactions in hubs. *Nat. Commun.* **9**, 5194 (2018).

40. S. S. Dima, M. A. Turner, H. G. Garcia, G. T. Reeves, Global maps of transcription factor properties reveal threshold-based formation of DNA-bound and mobile clusters. *Sci. Adv.* **12**, eady3909 (2026).

41. M. A. Turner, N. M. Gravina, B. Moretti, S. Dima, G. Martini, G. Reeves, H. G. Garcia, Novel fluorescent and photoconvertible fusions reveal dorsal activator dynamics. bioRxiv 653543 [Preprint] (2025). https://doi.org/10.1101/2025.05.12.653543.

42. J. P. Bothma, H. G. Garcia, S. Ng, M. W. Perry, T. Gregor, M. Levine, Enhancer additivity and non-additivity are determined by enhancer strength in the *Drosophila* embryo. *eLife* **4**, e07956 (2015).

43. E. Esposito, B. Lim, G. Guessous, H. Falahati, M. Levine, Mitosis-associated repression in development. *Genes Dev.* **30**, 1503–1508 (2016).

44. H. Deng, B. Lim, Shared transcriptional machinery at homologous alleles leads to reduced transcription in early *Drosophila* embryos. *Front. Cell Dev. Biol.* **10**, 912838 (2022).

45. R. Bouzignac, A. Palandri, A. Zine El Aabidine, T. Mangeat, T. Merle, M. Cazales, A. Trullo, C. Rouviere, V. Pimmett, M. Lagha, M. Suzanne, Spatio-temporal control of nuclear mechanotransduction during epithelial-to-mesenchymal transition. bioRxiv 672162 [Preprint] (2025). https://doi.org/10.1101/2025.08.25.672162.

46. P.-H. Holmqvist, A. Boija, P. Philip, F. Crona, P. Stenberg, M. Mannervik, Preferential genome targeting of the CBP co-activator by Rel and Smad proteins in early Drosophila melanogaster embryos. *PLOS Genet.* **8**, e1002769 (2012).

47. E. Tunnacliffe, J. R. Chubb, What is a transcriptional burst? *Trends Genet.* **36**, 288–297 (2020).

48. J. V. W. Meeussen, T. L. Lenstra, Time will tell: Comparing timescales to gain insight into transcriptional bursting. *Trends Genet.* **40**, 160–174 (2024).

49. S. Syed, Y. Duan, B. Lim, Modulation of protein-DNA binding reveals mechanisms of spatiotemporal gene control in early *Drosophila* embryos. *eLife* **12**, e85997 (2023).

50. Y. Sun, C.-Y. Nien, K. Chen, H.-Y. Liu, J. Johnston, J. Zeitlinger, C. Rushlow, Zelda overcomes the high intrinsic nucleosome barrier at enhancers during *Drosophila* zygotic genome activation. *Genome Res.* **25**, 1703–1714 (2015).

51. M. Kapoor, M. Mir, Simulation of transcription factor clustering in nuclei from molecular kinetics. bioRxiv 730171 [Preprint] (2026). https://doi.org/10.64898/2026.06.04.730171.

52. M. Mir, A. Reimer, M. Stadler, A. Tangara, A. S. Hansen, D. Hockemeyer, M. B. Eisen, H. Garcia, X. Darzacq, Single molecule imaging in live embryos using lattice light-sheet microscopy. *Methods Mol. Biol.* **1814**, 541–559 (2018).

53. A. S. Hansen, M. Woringer, J. B. Grimm, L. D. Lavis, R. Tjian, X. Darzacq, Robust model-based analysis of single-particle tracking experiments with Spot-On. *eLife* **7**, e33125 (2018).

54. S. Alamos, A. Reimer, C. Westrum, M. A. Turner, P. Talledo, J. Zhao, E. Luu, H. G. Garcia, Minimal synthetic enhancers reveal control of the probability of transcriptional engagement and its timing by a morphogen gradient. *Cell Syst.* **14**, 220–236.e3 (2023).

55. L. Dunipace, J. M. McGehee, J. Irizarry, A. Stathopoulos, The proximal enhancer of the snail gene mediates negative autoregulatory feedback in *Drosophila melanogaster*. *Genetics* **230**, iyaf058 (2025).

56. M. D. O'Connell, G. T. Reeves, The presence of nuclear cactus in the early *Drosophila* embryo may extend the dynamic range of the dorsal gradient. *PLoS Comput. Biol.* **11**, e1004159 (2015).

57. J. N. Wibisana, T. Inaba, H. Shinohara, N. Yumoto, T. Hayashi, M. Umeda, M. Ebisawa, I. Nikaido, Y. Sako, M. Okada, Enhanced transcriptional heterogeneity mediated by NF-κB super-enhancers. *PLOS Genet.* **18**, e1010235 (2022).

58. J. Jiang, M. Levine, Binding affinities and cooperative interactions with bHLH activators delimit threshold responses to the dorsal gradient morphogen. *Cell* **72**, 741–752 (1993).

59. R. Sayal, J. M. Dresch, I. Pushel, B. R. Taylor, D. N. Arnosti, Quantitative perturbation-based analysis of gene expression predicts enhancer activity in early *Drosophila* embryo. *eLife* **5**, e08445 (2016).

60. Y. T. Ip, R. E. Park, D. Kosman, E. Bier, M. Levine, The dorsal gradient morphogen regulates stripes of rhomboid expression in the presumptive neuroectoderm of the *Drosophila* embryo. *Genes Dev.* **6**, 1728–1739 (1992).

61. J.-W. Hong, D. A. Hendrix, D. Papatsenko, M. S. Levine, How the Dorsal gradient works: Insights from postgenome technologies. *Proc. Natl. Acad. Sci. U.S.A.* **105**, 20072–20076 (2008).

62. E. A. Leyes Porello, J. Wu, S. Fallacaro, E. Dreispiel Juan, R. T. Trudeau, J. A. Vidal, C. Nieto, A. Singh, M. Mir, B. Lim, Enhancer placement impacts transcriptional dynamics in *Drosophila* embryos. bioRxiv 670187 [Preprint] (2025). https://doi.org/10.1101/2025.08.14.670187.

63. C.-Y. Cho, P. H. O'Farrell, Stepwise modifications of transcriptional hubs link pioneer factor activity to a burst of transcription. *Nat. Commun.* **14**, 4848 (2023).

64. C. Fountas, T. L. Lenstra, Better together: How cooperativity influences transcriptional bursting. *Curr. Opin. Genet. Dev.* **89**, 102274 (2024).

65. T. L. Bailey, J. Johnson, C. E. Grant, W. S. Noble, The MEME suite. *Nucleic Acids Res.* **43**, W39–W49 (2015).

66. S. H. Keller, S. G. Jena, Y. Yamazaki, B. Lim, Regulation of spatiotemporal limits of developmental gene expression via enhancer grammar. *Proc. Natl. Acad. Sci. U.S.A.* **117**, 15096–15103 (2020).

67. J. A. Castro-Mondragon, R. Riudavets-Puig, I. Rauluseviciute, R. B. Lemma, L. Turchi, R. Blanc-Mathieu, J. Lucas, P. Boddie, A. Khan, N. Manosalva Pérez, O. Fornes, T. Y. Leung, A. Aguirre, F. Hammal, D. Schmelter, D. Baranasic, B. Ballester, A. Sandelin, B. Lenhard, K. Vandepoele, W. W. Wasserman, F. Parcy, A. Mathelier, JASPAR 2022: The 9th release of the open-access database of transcription factor binding profiles. *Nucleic Acids Res.* **50**, D165–D173 (2022).

68. T. Ferraro, E. Esposito, L. Mancini, S. Ng, T. Lucas, M. Coppey, N. Dostatni, A. M. Walczak, M. Levine, M. Lagha, Transcriptional memory in the *Drosophila* embryo. *Curr. Biol.* **26**, 212–218 (2016).

69. M. M. Harrison, X.-Y. Li, T. Kaplan, M. R. Botchan, M. B. Eisen, Zelda binding in the early *Drosophila melanogaster* embryo marks regions subsequently activated at the maternal-to-zygotic transition. *PLOS Genet.* **7**, e1002266 (2011).

70. T.-M. Fu, G. Liu, D. E. Milkie, X. Ruan, F. Görlitz, Y. Shi, V. Ferro, N. S. Divekar, W. Wang, H. M. York, V. Kilic, M. Mueller, Y. Liang, T. A. Daugird, M. J. Gacha-Garay, K. A. Larkin, R. C. Adikes, N. Harrison, C. Shirazinejad, S. Williams, J. L. Nourse, S.-H. Sheu, L. Gao, T. Li, C. Mondal, K. Achour, W. Hercule, D. Stabley, K. Emmerich, P. Dong, D. Drubin, Z. J. Liu, D. Clapham, J. S. Mumm, M. Koyama, A. Killilea, J. J. Bravo-Cordero, C. D. Keene, L. Luo, T. Kirchhausen, M. M. Pathak, S. Arumugam, J. K. Nunez, R. Gao, D. Q. Matus, B. L. Martin, I. A. Swinburne, E. Betzig, W. R. Legant, S. Upadhyayula, A multimodal adaptive optical microscope for in vivo imaging from molecules to organisms. bioRxiv 657494 [Preprint] (2025). https://doi.org/10.1101/2025.06.02.657494.

71. T.-L. Liu, S. Upadhyayula, D. E. Milkie, V. Singh, K. Wang, I. A. Swinburne, K. R. Mosaliganti, Z. M. Collins, T. W. Hiscock, J. Shea, A. Q. Kohrman, T. N. Medwig, D. Dambournet, R. Forster, B. Cunniff, Y. Ruan, H. Yashiro, S. Scholpp, E. M. Meyerowitz, D. Hockemeyer, D. G. Drubin, B. L. Martin, D. Q. Matus, M. Koyama, S. G. Megason, T.

Kirchhausen, E. Betzig, Observing the cell in its native state: Imaging subcellular dynamics in multicellular organisms. *Science* **360**, eaaq1392 (2018).

72. *Cudadecon: GPU Accelerated 3D Image Deconvolution Using CUDA* (Github); https://github.com/scopetools/cudadecon.

73. X. Ruan, M. Mueller, G. Liu, F. Görlitz, T.-M. Fu, D. E. Milkie, J. L. Lillvis, A. Kuhn, J. Gan Chong, J. L. Hong, C. Y. A. Herr, W. Hercule, M. Nienhaus, A. N. Killilea, E. Betzig, S. Upadhyayula, Image processing tools for petabyte-scale light sheet microscopy data. *Nat. Methods* **21**, 2342–2352 (2024).

74. M. Pachitariu, C. Stringer, Cellpose 2.0: How to train your own model. *Nat. Methods* **19**, 1634–1641 (2022).

75. A. Archit, L. Freckmann, S. Nair, N. Khalid, P. Hilt, V. Rajashekar, M. Freitag, C. Teuber, M. Spitzner, C. Tapia Contreras, G. Buckley, S. von Haaren, S. Gupta, M. Grade, M. Wirth, G. Schneider, A. Dengel, S. Ahmed, C. Pape, Segment anything for microscopy. *Nat. Methods* **22**, 579–591 (2025).

76. S. Fallacaro, A. Mukherjee, E. Gamauf, M. Mir, Quantifying transcription factor enrichment at target loci in live embryos. *Methods Mol. Biol.* **2923**, 119–141 (2025).

77. M. Pachitariu, M. Rariden, C. Stringer, Cellpose-SAM: Superhuman generalization for cellular segmentation. bioRxiv 651001 [Preprint] (2025). https://doi.org/10.1101/2025.04.28.651001.

78. A. Heckert, *Quot: A Simple Single Molecule Tracking Pipeline with a Graphic User Interface for Quality Control* (Github); https://github.com/alecheckert/quot.

79. A. S. Hansen, I. Pustova, C. Cattoglio, R. Tjian, X. Darzacq, CTCF and cohesin regulate chromatin loop stability with distinct dynamics. *eLife* **6**, e25776 (2017).

80. K. J. Brennan, M. Weilert, S. Krueger, A. Pampari, H.-Y. Liu, A. W. H. Yang, J. A. Morrison, T. R. Hughes, C. A. Rushlow, A. Kundaje, J. Zeitlinger, Chromatin accessibility in the

Drosophila embryo is determined by transcription factor pioneering and enhancer activation. *Dev. Cell* **58**, 1898–1916.e9 (2023).
